# Effectiveness and theory-based evaluation of a personalised digital intervention (EviBody^®^) for healthy and sustained lifestyle behaviours and well-being among adults: Study protocol for a real-world quasi-experimental study

**DOI:** 10.1371/journal.pone.0333201

**Published:** 2025-10-07

**Authors:** Jenny Rossen, Therese Anderbro, Susanne Andermo, Patrick Bergman, Maria Hagströmer, Mattias Jacobsson, Unn-Britt Johansson, Lena Kallings, Amanda Lönn, Callum Regan, Philip von Rosen

**Affiliations:** 1 Department of Health Promoting Science, Sophiahemmet University, Stockholm, Sweden; 2 Department of Psychology, Stockholm University, Stockholm, Sweden; 3 Department of Neurobiology, Care Sciences and Society, Karolinska Institutet, Stockholm, Sweden; 4 Department of Physical Activity and Health, The Swedish School of Sport and Health Sciences, Stockholm, Sweden; 5 eHealth Institute, Linnaeus University, Kalmar, Sweden; 6 Academic Primary Care Center, Region Stockholm, Stockholm, Sweden; 7 Södertörns University, Stockholm, Sweden; 8 The Research Institute of Sweden, RISE, Stockholm, Sweden; RAK Medical and Health Sciences University, UNITED ARAB EMIRATES

## Abstract

**Background:**

Digital interventions offering behaviour change support are warranted to prevent and treat non-communicable diseases, and have been evaluated rigorously in controlled settings. Effectivenss, factors influencing the uptake of scaled-up interventions—such as reach, received dose, usability and acceptability— and predictors and mediators of efficiency are rarely explored in research. The study described herein aims to evaluate the effectiveness of a personally tailored digital intervention (the app EviBody^®^), intended to support healthy and sustained lifestyle behaviours among the adult population, on well-being and behaviour change. Further aims are to explore context and uptake factors, predictors and mediators for behaviour change over 24 months.

**Methods:**

This is a real-world study, employing a quasi-experimental design and a process evaluation. EviBody^®^ will be marketed and managed by its owner. A four-armed design will allow for comparison between three levels of intervention (basic, standard and premium) and a control group. Adults who sign up for the app will be invited to the research study including sharing app data and answering questionnaires at 0, 1, 3, 6, 12, 18, and 24 months. Study start is Autumn 2025. Controls (n = 200 to evaluate the primary endpoint well-being at 6 months) will be recruited through advertisements on social media and asked to answer the same questionnaires at 0 and 6 months provided by email. For predicting and mediating analyses the intention is to recruit 1500 app users. Well-being (measured with the WHO-5 Well-Being Index), goal achievement, physical activity, eating habits, mental health, mediators (motivation, self-efficacy, and perceived barriers), and demographics will be self-reported. Uptake will be collected using analytics and ratings of usability and acceptability, and described by demographics. Mixed models for repeated measures and structural equation modelling will be employed for data analysis.

**Discussion:**

Besides evaluating the effectivenss of a digital intervention, this study also applies a theory-based evaluation to understand which mediators are effective, for whom they are effective, and the specific conditions under which they are most beneficial.

**Trial registration:**

ClinicalTrials.gov, ID: NCT05973383 on 8 July 2023.

## Introduction

Noncommunicable diseases (NCDs), such as cardiovascular disease, diabetes, cancer, and depression are the leading causes of death and morbidity globally and are expected to account for 90% of all deaths by 2048 [1]. Reducing premature mortality from NCDs through prevention and treatment, and promoting mental health and well-being are targets in the sustainable development goal 3.4, of Agenda 2030 [2]. Unhealthy lifestyle behaviours such as poor dietary habits and physical inactivity are leading risk factors for NCDs [3] and are associated with low well-being [4–6]. Promoting healthy lifestyle behaviours is central to preventing and treating NCDs. Longitudinal studies have shown that improved lifestyle behaviours may enhance well-being [4,7], but intervention research studying the effects of lifestyle change on well-being among adults is limited.

Changing behaviour and maintaining a new behaviour can be challenging. When properly designed, digital interventions providing behaviour change techniques have the potential to improve short-term adherence to improved eating habits [8], physical activity levels [8–11], and health outcomes (e.g., anthropometrics [12–14], stress levels [14] and clinical outcomes [12]) in controlled settings. Evidence suggests that behaviour change techniques facilitating self-regulation [15–17], personalisation of goals, and opportunities for social interaction [16,17] increase the efficacy of digital interventions targeting physical activity and eating behaviours. Interventions including a counselling component versus simpler interventions (e.g., self-monitoring of behaviour) have been reported to have an additional effect on behaviour change [11]. However, the form for counselling varies among studies (individual or group counselling, delivered face-to-face, via emails, phone, or digital), and the evidence is limited regarding the effectiveness of digital counselling. More research evaluating separate intervention components is needed to establish best practices for digital behaviour change interventions [8,11,18].

Research has until now focused on short-term controlled efficacy trials and up-scaled long-term (> 12 months) implementation studies are rare [11,13,14,18]. Real-world trials are required to establish if digital interventions are effective for behaviour change outside the controlled research setting [18–20]. Major challenges of digital interventions are high abandonment rates [21,22] and difficulties reaching population groups most in need [14]. Factors influencing the uptake of digital interventions need to be understood and overcome [10,22,23]. Yet, uptake factors such as who is reached and who has engaged with the intervention, the dose of the intervention that participants actually receive or engage with, usability and acceptability as well as predictors and mediators of efficiency are rarely studied and require further investigation [8,10,12,24,25]. Theory-based evaluations exploring and theorising how intervention works are essential to enhance the impact and translation of behaviour interventions [26,27].

Based on the need to find effective solutions supporting healthy lifestyle behaviours for the prevention and treatment of NCDs in the general population, this research project was initiated in 2020. A digital intervention was developed through a co-development process considering the perspectives of product developers, researchers, end users, and healthcare professionals. It was decided to deliver the intervention as an app, named LongLife Active (the name was changed to EviBody^®^ in 2024). A prototype of the app was evaluated and further refined according to feedback from users [28,29]. EviBody^®^ is now available at three membership levels in the Swedish language in App Store and Google Play. The launching of the app provides an opportunity to study the effectiveness of a digital intervention in a real-world situation. In addition, it provides an opportunity for theory-based evaluation; to explore the uptake and the interaction of mechanisms and context of a digital intervention. Here we describe the development and content of the intervention and provide a detailed plan for evaluation following the SPIRIT 2013 checklist for interventional trials (S1 File). The study was registered at ClinicalTrials.gov with Identifier: NCT05973383 on 8 July 2023.

## Materials and methods

### Aims and hypothesis

The primary aim of this study is to evaluate the effectiveness of three membership levels of the digital intervention (EviBody^®^) in supporting healthy lifestyle behaviours and improving well-being among the adult population. Our primary hypothesis is that users who engage with EviBody^®^ will improve their lifestyle behaviours (user’s choice of physical activity, eating habits, or mental health) and thereby improve well-being compared to the control group. With the purpose to evaluate programme theory, secondary aims are to explore the context and uptake of the digital intervention and to explore predictors and mediators for behaviour change.

### Objectives

The study includes several specific objectives that will be targeted in sub-studies.

1 **Effectiveness**
  To evaluate the effectiveness of three levels of digital support on well-being, at 6 months, among adults using the intervention compared to a control group from the general adult population (primary end-point).
  To explore the effectiveness of the digital intervention on achieving self-identified goals, improving eating and physical activity habits, mental health, and well-being over a 24-month period.
2 **Process evaluation**
  To describe the context, uptake factors (including who is reached and who has engaged with the intervention, the intensity of the engagement, and its usability and acceptability), and any adaptations made to the digital intervention, over a 6-month period.
  To explore engagement levels of the digital intervention and adherence to behaviour change, over a 24-month period.
3 **Predictors, moderators and mediators**
  To explore whether sociodemographic factors serve as predictors of intervention effectiveness over a 24-month period.
  To explore whether sociodemographic factors serve as moderators of intervention effectiveness over a 24-month period.
  To examine whether engagement, motivation, self-efficacy, and perceived barriers mediate the associations between sociodemographic characteristics and changes in self-identified goals, eating and physical activity habits, mental health, and well-being over a 24-month period.

### Designs and setting

**Fig 1:**
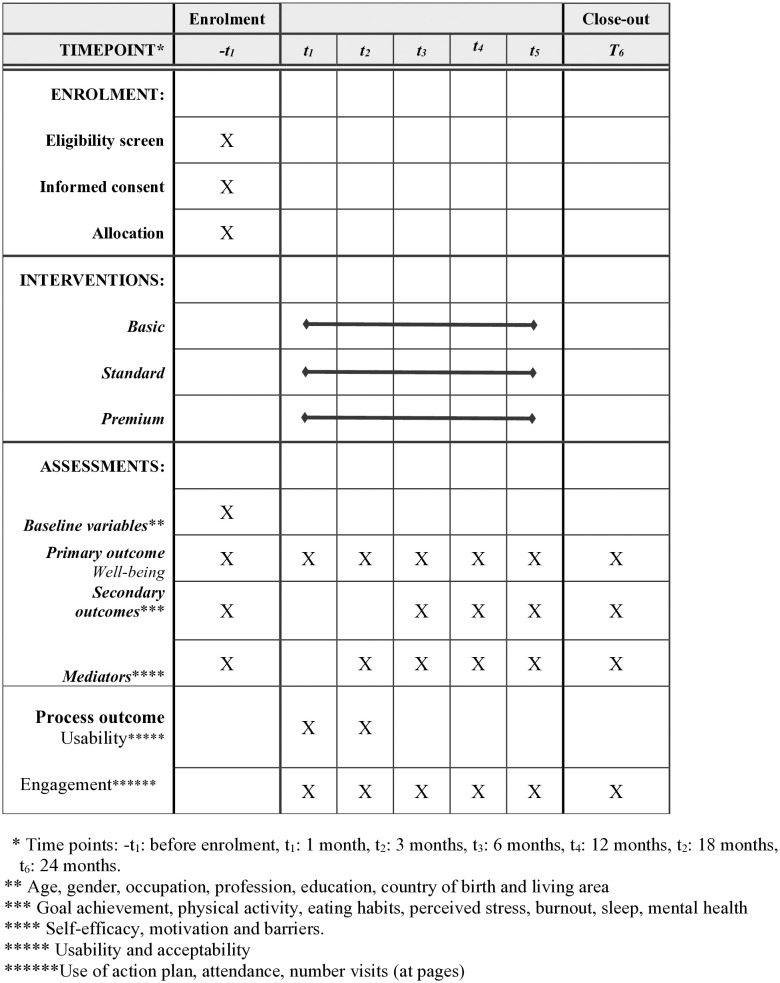
Schedule of enrolment, interventions, and assessments.

The primary study is a quasi-experimental study with a non-randomised control group. A four-armed design will allow for comparison between three levels of intervention (basic, standard and premium) and a control group. Fig 1 shows a SPIRIT schedule of enrolment, interventions, and assessments.

**Fig 2:**
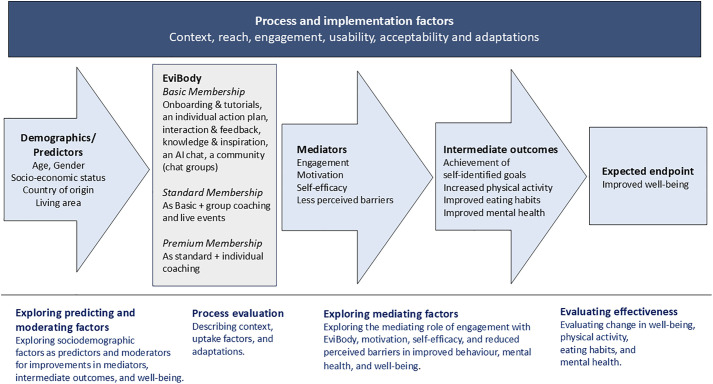
Logic model. The logic model visualises the factors expected to influence the effectiveness of the digital intervention on the endpoint well-being; process and implementation factors, demographics/predictors, the intervention components of EviBody^®^, and intended mediators of the intervention. Outcomes are pictured as behavioural intermediate outcomes and the endpoint well-being. At the bottom are planned studies briefly described.

A process evaluation is planned to explore the context, uptake factors, and adaptations of the digital intervention following the Medical Research Council framework for developing and evaluating complex interventions [26,30]. The project will also collect data to explore patterns of engagement, behaviour change, predictors, and mediating factors longitudinally. Fig 2 shows a logic model for the intervention and planned studies.

### Description of materials and context

This is a real-world study and the digital intervention (the app EviBody^®^) will be marketed and managed by its owner LongLife Active AB (Limited Company), Sweden. The intention is to reach individuals requiring lifestyle change on a national level. Employers, benefits platforms, healthcare services, pharmacies, and social media will be targeted channels. Employers may apply for the health-promoting subsidy provided by the Swedish government when employees sign up for the app. Healthcare patients, who receive physical activity on prescription by their care provider, will have a reduced membership fee for the app. Individuals who sign up for the app will be informed about the research study and are invited to provide consent in the app. Allocation to intervention arm is by preference (chosen membership level of the app) and takes place at enrolment. Being a research participant includes answering seven questionnaires during two years and allowing researchers to extract data from app analytics. Controls will be recruited through advertisements on social media. After a control participant shows interest in participating in the study, information about the study is then sent by email, together with a link to the web-based questionnaire. Being a control participant includes answering a questionnaire two times; at the start and again after 6 months. Consent is collected upon answering the first questionnaire.

The study was registered at ClinicalTrials.gov, ID: NCT05973383 on 8 July 2023 and ethical approval was obtained from the Swedish Ethical Review Authority, Dnr 2023-06246-01 on January 30 2024. Expected recruitment period is 01/10/2025–31/12/2026 and data collection for all substudies is estimated to be completed 31/12/2028. Results from the primary analyses are expected to be reported in Autumn 2027.

Table 1 shows the inclusion and exclusion criteria for the intervention and control participants.

**Table 1:** Inclusion and exclusion criteria.

	Intervention arms	Control arm
Inclusion criteria	Adults (≥18 years) who use a smartphone and sign up for the app EviBody^®^ using electronic identification, and consent to the research study.	Adults (≥18 years) registering interest via social media and consenting to partake.
Exclusion criteria	Individuals who are discharged from the app due to refracting the terms of the service. Is currently using a digital product that is explicitly designed to support behaviour change. Users scoring ≥70 on well-being will be excluded from the primary analyses on well-being. Users who appear as friends, colleagues, or family with anyone in the research or owner group will be excluded.	Is currently using a digital product that is explicitly designed to support behaviour change or is found to be a user of the app (EviBody^®^). Subjects scoring ≥70 on well-being will be excluded from the primary analyses on well-being. Subjects who appear as friends, colleagues, or family with anyone in the research or owner group will be excluded.

### The development of the digital intervention

**Fig 3:**
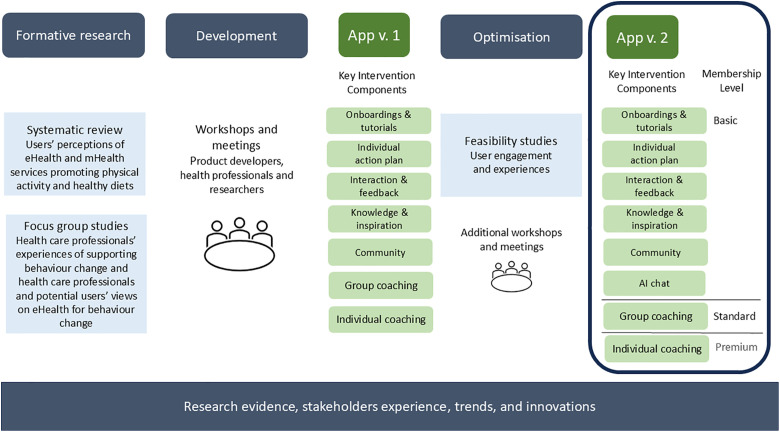
Illustration of the co-development process. The illustration shows the intervention development through formative, development and optimisation phases, resulting in key intervention components. Two feasibility studies were undertaken as part of the optimisation phase. The optimisation phase resulted in refinements of the app, structuring the support based on the stages of change model, and the development of an AI chat. Throughout the process, former research, stakeholders’ experience, trends in society, and innovations were discussed and applied.

The project is guided by the framework for planning interventions “The person-based approach to planning, optimising, evaluating and implementing behavioural health interventions” [31] and the Medical Research Council framework for developing and evaluating complex Interventions [26]. The project started in 2020 with an intervention planning phase. Fig 3 shows an overview of the development process.

Firstly, in the formative phase, two qualitative studies explored users’ and healthcare professionals’ perspectives on support for physical activity [32,33]. Further, a literature review was undertaken to summarise users’ views on eHealth and mHealth services promoting physical activity and healthy diets [34]. These studies showed, for example, that users desired a digital intervention to be dynamic, and integrated with other services and for the interface design and reminders to be customisable [32,34]. In 2021, the digital intervention was developed using a co-development approach including 11 researchers, 5 product developers, and 5 healthcare professionals. In total, nine workshops were conducted, along with numerous short meetings in smaller groups in between the workshops. Core guiding principles for the development and the uniqueness of this intervention were decided as: sustained motivation, enhanced engagement, and personalisation. At this stage, key features for the intervention were decided upon and developed in a prototype of the app.

Secondly, in the intervention optimisation phase, two feasibility trials were undertaken and used to guide the refinement of the digital intervention. In June-August 2022, the first version of the app was tested by 55 users for 12 weeks to investigate usability, acceptability, engagement and optimisation in a mixed-methods approach [28]. During February-April 2023, 26 individuals with type 2 diabetes tested the app for 1 month and 10 agreed to be interviewed after the test period to evaluate their perceptions of the app [29]. The users’ engagement and feedback, from these feasibility studies, were used to improve the intervention, especially the interface design of the action plan (the feature to set and register goals). The action plan was perceived as rather confusing, therefore it was decided to develop an AI chat to assist with this. Health professionals with expertise and experience in health coaching and motivational interviewing developed phrases and constructed dialogues for the chat. It was also decided to tailor functions, reminders, and inspiration according to the users’ stage of change, based on the Transtheoretical Model of Change [35,36]. An additional workshop was conducted in November 2023, including researchers, product developers, and healthcare professionals, to discuss and further develop the behaviour change process according to the stages of change. The two feasibility studies also tested the questionnaires to be used in this trial and the process for data collection, as part of scientific feasibility. All along the development, research evidence, stakeholders’ experiences, and trends and innovations in society were considered and discussed.

### Intervention

The digital intervention includes three core subjects 1) Healthy eating, 2) Physical activity, and 3) Mental health. The user can engage in one, two, or all three core subjects. Healthy eating is based on the Nordic Nutrition Recommendations 2023 [37] and physical activity is based on the Swedish guidelines for physical activity [38]. The subject mental health is named Mental balance and includes habits for recovery, mental calmness, digital disconnection, and sleep. The content, layout and navigation can be viewed in S1 Video.

Key intervention components encompass *onboarding and tutorials, individual action plan, interaction and feedback, knowledge and inspiration, AI-chat, and community.* The included intervention components and applied behaviour change techniques are presented in S2 File. The digital intervention is assisted by AI to direct the support to the individual timely and based on each user’s goals, readiness for change, preferences, capabilities, and progress. Users may sign up for one of three membership levels. The first level (Basic) includes the mentioned key intervention components. The second level (Standard) has an expanded community component *Group coaching* including live events and group coaching. The third level (Premium) additionally includes the component *individual coaching*.

The intervention component *Onboarding and tutorials* is intended to introduce the user to the app and to give an overview of the included components through short texts, animations, and videos. The component *Individual action plan* involves imagining future outcomes, goal setting, and the possibility of setting and registering daily or weekly activities. *Interaction and feedback* include feedback on behaviour, rewards and encouragement, educational and inspirational prompts to inform the user about realistic goals and actions to adopt a healthy and sustained lifestyle. It also includes reminders about planned activities and booked live events and coaching sessions. The component *Knowledge and inspiration* includes short articles and videos with inspiration, facts, and tips for behaviour change, healthy eating, physical activity, and improved mental health, all based on guidelines and research evidence. In addition, this component includes a library with >100 easy-to-cook and healthy recipes, > 200 video-recorded home-based exercises of various degrees of difficulty and intensity, and meditation exercises. An *AI chat* is included as a core component to offer individualised support and guidance concerning the user’s action plan and goals. The *Community* is a digital forum for the user to get social support from others in the presence of health coaches. The health coaches publish 3–5 post/week to inspire and educate the users about behaviour change and a healthy lifestyle using materials from the library and encourage social interaction. The users can react and comment, e.g., ask a question, encourage other group members, share a success or propose a challenge, and vote on the posts published by the health coach. For disease-specific groups (e.g., “diabetes-group”, “obesity-group”) specialised nurses will moderate the group in collaboration with the health coach.

Users with Standard and Premium memberships can also join digital weekly live events, e.g., group training or meditation with a health coach, group coaching sessions, webinars held by a health coach or a researcher, and, in geographic areas with several users, physical meetups.

Premium membership also includes *Individual coaching*. The number of individual health consultations is one for a one-month subscription three for a 3-month subscription, four for a 6-month subscription, and six for a 12-month subscription. A requirement to become a health coach, and lead individual- and group counselling in digital video meetings, is having a bachelor’s degree in health education. The coaches are also trained in how to use the app during a mandatory introduction course before operating the app.

With a simple design, pictures, short texts, and video instructions, the app is customised for people with low health literacy, low skills in the Swedish language, or with low digital literacy. Online technical support is available for all users and health coaches, both through contacting the support and through the AI chat.

### Measures

Data will primarily be collected through the app as self-reported answers to questionnaires and as app analytics. Table 2 shows an overview of time points and instruments used for data collection. Below is a brief description of the respective outcomes and instruments that will be used to collect data. All instruments are well-established, valid, and reliable, and were also selected for their relevance to self-monitoring and behavioural feedback for users.  Two weekly reminders will be sent for each questionnaire, both to control and intervention participants. The questionnaires will be provided in the Swedish language.

**Table 2:** Overview of time points and instruments used for data collection.

Variable	Instrument	Time point (month)						
		0	1	3	6	12	18	24
**Demographics and predictors**								
Age	–	✓						
Gender	–	✓						
Occupation	–	✓						
Profession	–	✓						
Education	–	✓						
Country of birth	–	✓						
Living area	–	✓						
**Well-being and mental health**								
Well-being	WHO-5 Well-being index	✓	✓	✓	✓	✓	✓	✓
Perceived stress	The Karolinska Exhaustion Disorder Scale (KEDS)	✓		✓	✓	✓	✓	✓
Burnout	The Oldenburg Burnout Inventory (OLBI)	✓		✓	✓	✓	✓	✓
Sleep	Self-reported number of hours at sleep	✓		✓	✓	✓	✓	✓
	Sleep diary^a^	✓		✓	✓	✓	✓	✓
	Insomnia Severity Index (ISI)	✓		✓	✓	✓	✓	✓
Mental health	–	✓		✓	✓	✓	✓	✓
**Goal achievement**								
Confidence in reaching the goal	–	✓		✓	✓	✓	✓	✓
Days/week the self-identified goal was met during the past month	Analytics	✓	✓	✓	✓	✓	✓	✓
**Mediators**								
Self-efficacy	The General Self-Efficacy Scale (S-GSE)	✓		✓	✓	✓	✓	✓
Motivation	Readiness to Change Questionnaire (RTCQ)	✓		✓	✓	✓	✓	✓
Barriers	CDC Road to Health Barriers	✓		✓	✓	✓	✓	✓
**Lifestyle habits**								
Physical activity	Daily number steps from the app^b^	✓	✓	✓	✓	✓	✓	✓
	Questions from the National Board of Health and Welfare and questions on active transport and physical activity at work.^b^	✓			✓	✓	✓	✓
Eating habits	Questions from the National Board of Health and Welfare and additional questions on plant-based food.^c^	✓			✓	✓	✓	✓
**Usability**								
Usability	System Usability Scale (SUS)		✓	✓				
Acceptability	Mobile Application Rating Scale (MARS)		✓	✓				
**Engagement**								
Use of action plan	Analytics		✓	✓	✓	✓	✓	✓
Number of visits at core intervention components	Analytics		✓	✓	✓	✓	✓	✓

^a^Applicable only to subjects who have chosen sleep for behaviour change.

^b^Applicable only to subjects who have chosen physical activity for behaviour change.

^c^Applicable only to subjects who have chosen healthy eating for behaviour change.

#### Primary outcome measure.

Well-being will be assessed with the WHO-5 Well-Being Index [39]. The WHO-5 comprises the following five items: being cheerful and in good spirits; being calm and relaxed; feeling active and vigorous; feeling fresh and rested when waking up in the morning; and having an interest in day-to-day activities, during the last two weeks. Six response alternatives are scored from 5 (All of the time) to 0 (At no time). The total raw score which ranges from 0 to 25 is multiplied by 4 to calculate the final score. The final score ranges from (0) the worst imaginable well-being to (100) the best imaginable well-being.

#### Secondary outcome measures.

**Goal achievement:** Reaching the self-identified goals will be evaluated monthly by collecting information on number of days the self-identified goals were met. Confidence in reaching the goals will also be asked for on a scale of 1 low confidence to 6 high confidence.

**Physical activity and sedentary behaviour:** Physical activity and sedentary behaviour will be assessed through self-reported questions and step counts. Self-reported questions for physical activity include asking participants regarding their weekly time performing physical activities at moderate intensities such as walking, biking, and gardening and at high to vigorous activities such as running, aerobics, or ball sports. The responses are categorised as 0 (0 p), less than 30 (1 p), 30–60 (2 p), 60–90 (3 p), 90–150 (4 p), 150–300 (5 p) or more than 300 minutes (6 p) for moderate intensity activities and 0 (1 p), less than 30 (4 p), 30–60 (6 p), 60–90 (8 p), 90–120 (10 p), or more than 120 minutes (12 p) for vigorous intensity activities. The points are summed to a scale ranging from 1–18 [40]. Muscle-strengthening activities are assessed via a question asking how many times per week muscle-strengthening activities are performed. Responses range from 0, 1, 2, or more than 2 times. Daily steps are assessed with the question “How many steps do you take on a normal day?” with responses categorised as; 0–1500, 1500–2500, 2500–4000, 4000–5000, 5500–7000, 7000 steps or more or Do not want to/can not answer. Sedentary behaviour is assessed as how much one is sitting on a normal day with responses categorised as; pretty much all day, 13–15, 10–12, 7–9, 4–6 hours, or not at all [41]. Active transport is asked with the question “Do you usually walk/bike to get to and from places? If yes, “How much time in a week do you spend walking/biking to get to and from places? Sum up all time” [42]. Physical activity at work is assessed by asking participants if they have physically demanding work tasks at light level (e.g., brisk walk, carrying light loads as stockroom, store or care work) and at a vigorous level (carrying or lifting heavy loads, digging or construction work that causes heavy breating) and how much time in a week is spent in such activities. Responses for each respective question for active transport and physical activity at work are; 0, less than 30, 30–60, 60–90, 90–150, 150–300 or more than 300 minutes. Daily number of steps will also be obtained from the smartphone (Health Connect for Android and Apple Health for iOS) and collected via the app. Only participants who have chosen to change their physical activity habits will answer these questions, to reduce the burden on participants.

**Eating habits:** Eating habits will be assessed with questions regarding how often (seldom, weekly, daily, or several times daily), one eats food from different food groups. The response scores ranges from 0–4 depending on regularlity of consumption: seldom, weekly, daily, or several times daily. 1) fruits and berries (0–4), 2) vegetables (including roots and tubers) (0–4), 3) fish and seafood (0–3), 4) nuts, seeds (0–2), 5) vegetable oils (0–2), 6) sugar-sweetened beverages (0–3, less scores higher), 7) alcoholic beverages (0–3, less scores higher), 8) unhealthy snacks (candy, pastries, ice-cream, crisps, salty nuts) (0–3, less scores higher), 9) ready-made meals or fast food (0–3, less scores higher), 10) red meat and processed meat (0–3, less scores higher), 11) legumes or ready-made meals based on legumes (0–2), and 12) whole-grain products (0–3). A sum score, but also change in respective eating behaviour will be used to assess change in eating habits. These questions are based on Swedish food frequency questionnaires [43,44] with added questions to match the recent Nordic Nutrition Recommendations 2023 [37]. Only participants who have chosen to change their dietary habits will answer these questions, to reduce the burden on participants.

**Mental health:** Mental health will be assessed as the ability to recover, perceived stress, burnout, and sleep. Four questions have been developed, specifically, for this study to assess ability to recover: Do you dedicate time for recovery in your everyday life? Do you dedicate time to digitally disconnect from your everyday life? Do you find that you can relax from thoughts of anxiety and stress to feel mental peacefulness? Do you feel recovered after a night’s sleep? The responses range from no, never (0), no, seldom (1), ye, often (2), to yes, regularly (3). A sum score, as well as change in each respective behaviour will be used to assess change in mental health outcomes.

Perceived stress will be assessed with the Karolinska Exhaustion Disorder Scale (KEDS), a self-rating scale for stress-induced exhaustion disorder symptoms the past two weeks [45]. KEDS includes nine items covering: 1) ability to concentrate, 2) memory, 3) physical stamina, 4) mental stamina, 5) recovery, 6) sleep, 7) hypersensitivity to sensory impressions, 8) experience of demands, and 9) irritation and anger. Responses to each item are made on a seven-point scale (0–6) and the sum score (0–54) will be used as an outcome. Higher values reﬂect more severe symptoms [45].

Burnout will be assessed with the Oldenburg Burnout Inventory (OLBI) [46]. The OLBI measures two dimensions of burnout; exhaustion and disengagement, with eight items to each dimension. Exhaustion refers to general feelings of emptiness, overtaxing from work, a strong need for rest, and a state of physical exhaustion. Disengagement refers to distancing oneself from the object and the content of one’s work and to negative, cynical attitudes and behaviours toward one’s work in general. There is no time aspect for OLBI. Responses range from: (1) Strongly disagree to (4) Strongly agree. Responses to each item are made on a four-point scale (1–4) and the sum score (32 for exhaustion respective disengagement) will be used as an outcome. Higher values reﬂect more severe symptoms [47].

Sleep will be assessed via self-reported hours of sleep on a usual night and with the Insomnia Severity Index (ISI) [48]. The ISI includes seven items assessing difficulties with 1) sleep-onset, 2) sleep maintenance (nocturnal), 3) sleep maintenance (early wake-up), 4) satisfaction with current sleep pattern, 5) interference with daily functioning, 6) noticeability of impairment attributed to the sleep problem, and 7) degree of worry caused by the sleep problem. The time interval for responses refer to the last two weeks. Responses for each item range from: (0) not at all to (4) extremely. The total score ranges from 0 to 28, a higher score indicates more severe insomnia. The cut off points that will be applied are: 0–7 = no clinical insomnia difficulties, 8–14 = mild insomnia, 15–21 = moderate insomnia, 22–28 = severe insomnia [48]. Data from a sleep diary will be collected from participants who have chosen to change their sleeping habits.

#### Process evaluation measures.

**Reach:** Demographics will be collected to explore reach, with a questionnaire at the start of the study including questions regarding participant’s age (years), gender (male, female, other, don´t want to answer), occupational status (working, on sick leave, unemployed, retired, student, leave on absence, parental leave, other), profession (open answer), education (primary school, high school, university), country of birth (open answer) and living area (area code and city). Information about how the participant received information about EviBody^®^ will also be collected.

**Engagement and intervention dose:** Data on engagement (user interaction with the app) will be collected as app analytics, aggregated per week and described for the core intervention components: *Onboarding and tutorials*, *Individual action plan, Interaction and feedback, AI chat, Community, Group coaching* and *Individual coaching*. The intervention dose will be estimated using days of app use throughout the intervention period.

**Adaptations:** Qualitative data from the support chat, and from dialogues with health coaches and staff maintaining the app, will be collected continuously. Records regarding notable adaptations and changes made to the intervention will be tracked in a monitoring plan.

**Usability and acceptability:** The perceived usability of the app will be assessed via the System Usability Scale (SUS) [49]. The SUS is a ten-item scale giving a global assessment of usability with five responses, ranging from: (1) Strongly disagree to (5) Strongly agree. The scores are multiplied by 2.5 and the total score is summed (scores range from 0–100). Acceptability will be assessed using the Mobile Application Rating Scale (MARS) [50]. MARS assesses app quality with 19 items covering four dimensions: engagement, functionality, aesthetics, and information. All items are rated on a 5-point scale from: (1) Inadequate to (5) Excellent. The mean score will be used to assess app quality. Additionally, three questions to assess subjective app quality will be used separately: 1) Would you recommend this app to someone who might benefit from it? Response scale: (1) not at all to (5) Definitiely. 2) How many times do you think you would use this app in the next 12 months if it feels relevant to you? Response scale: (1) None to (5) >50. 3) What is your overall star rating of the app? Response scale (1) One of the worst apps I’ve used to (5) One of the best apps I’ve used.

#### Measures of mediating factors.

**Motivation:** Motivation will be assessed through the readiness to change questionnaire (RTCQ), modified to suit behaviours relating to physical activity, healthy eating, and mental health [36]. The questionnaire include 12 items assesing three stages of change: precontemplation: no thoughts and no interest in changing behaviour, contemplation: no current behaviour but considering adopting regular behaviour within the near future, action: making active choices and performing the behaviour regularly. Each item is scored and a continuous readiness total score 0–24 will be used. There is no time aspect for RTCQ.

**Self-efficacy:** Self-efficacy will be evaluated using the Swedish version of the General Self-Efficacy scale (S-GSE) [51]. When answering the questions the participants are asked to regard their intended behaviour change and goals. This Swedish version includes 10 items: 1) I can always manage to solve difficult problems if I try hard enough, 2) If someone opposes me, I can find the means and ways to get what I want, 3) It is easy for me to stick to my aims and accomplish my goals, 4) I am confident that I could deal efficiently with unexpected events, 5) Thanks to my resourcefulness, I know how to handle unforeseen situations, 6) I can solve most problems if I invest the necessary effort, 7) I can remain calm when facing difficulties because I can rely on my coping abilities, 8) When I am confronted with a problem, I can usually find several solutions, 9) If I am in trouble, I can usually think of a solution, 10) I can usually handle whatever comes my way. Four responses for each question range from (1) Not at all true to (4) Exactly true. The total score ranges from 10–40, a higher score indicating more self-efficacy. There is no time aspect for the S-GSE scale.

**Barriers:** Barriers to undertaking healthy lifestyle behaviours will be evaluated using the Barriers to being active quiz [52]. The quiz was developed for barriers to physical activity but has been adapted for this study to assess barriers to consuming a healthy diet and achieving mental health. The instrument explores reasons for not performing the specified behaviour by asking whether one agrees with 21 statements about common barriers. The responses range from: (0) very unlikely to (3) very likely. An open-ended question is also added to capture additional barriers. There is no time aspect for the Barriers to being active quiz.

### Statistical analysis

Change in the primary outcome well-being and the intermediate outcomes physical activity, eating habits, and mental health will be analysed between time and across groups using mixed models for repeated measures. The advantage of mixed modeling is that it can handle unbalanced and missing data. The models will include intervention level and time. The statistician will be blinded to intervention allocation. Interaction effects will also be tested between intervention level and sociodemographic factors. As a sensitivity analysis to assess the robustness of the primary findings and address potential self-selection bias, we will conduct 1:1 propensity score matching, pairing each control participant with one intervention participant from each respective intervention arm [53]. Structural equation modeling will explore the relationship between sociodemographic factors (age, gender, occupational status, profession, education, country of birth, and living area) and user engagement with behaviour change and change in well-being. Structural equation modeling will also be applied to explore motivation, self-efficacy, and less perceived barriers as potential mediators of reaching self-identified goals, change in physical activity, eating habits, mental health, and well-being. Fig 1 shows a logic model for the project with the anticipated predictors, mediators, and intermediate and primary outcomes and S3 File outlines theoretical models for the planned prediction, moderation and mediation analyses. The threshold for statistical significance will be set at p < 0.05.

#### Sample size calculation and estimation.

A change of 10−20 points in the WHO-5 has been proposed as a clinically relevant change for well-being [54,55]. In up-scaled interventions, 60% lower effects are expected [56]. Therefore, a difference of 12 points in the WHO-5 score between the intervention groups and the control group at 6 months, as well as between baseline and 6 months, will be used as the minimum clinically relevant change.

To estimate the required sample size for detecting a significant treatment effect, power calculations using the simr package in R (version 4.3.1) (R Core Team, 2023) were performed. The power analysis targeted a significance level of α = 0.05, a within-subject standard deviation of 10, and a power of 80% to detect a clinically meaningful effect size of 12 points, for the primary outcome, well-being, at 6 months, with a standard deviation of 17.1 [57]. A linear mixed model was specified with a fixed effect for the treatment group and a random intercept for subjects, assuming an unstructured covariance structure for the repeated measures to flexibly model the variance and covariance between observations. Based on these parameters, the power calculation indicated that a sample size of 40 participants per group was required to achieve the desired power to detect a difference of 12 points in the WHO-5.

Participants with a well-being score ≥70 are not expected to significantly improve their well-being and will be excluded from the analyses [54]. In the pilot study, 21% of participants reached 70 points at baseline and we expect the same proportionin this study. The expected drop-out rate due to withdrawal and loss to follow-up at six months is estimated to be 75%. Taking into account these exclusions, and the fact that the intervention arm (membership level) is self-selected, we estimate that at least 810 intervention participants will need to be recruited to ensure at least 40 participants with pre and post measurements are in each intervention arm for the primary analyses. Furthermore, we estimate that we need to recruit 200 control participants to ensure that at least 40 participants will have pre and post measurements.

The secondary outcomes are exploratory due to the person-centred design of the intervention. The subjects will set individual goals (e.g., reducing weekly servings of red meat, increase fiber intake, increase number of daily steps, or start with muscle strengthening activities) and possibly change goals during the intervention. The current research is not immense enough to guide a decision for minimal expected effects when individual goals are set. A larger sample size than required for the primary endpoint, well-being, is desired. Similarly, the analyses of predictors and mediating factors require a large sample size. We therefore plan to include 1500 participants in the cohort, but will continuoulsy evaluate the number of participants needed for each analysis.

### Ethical considerations

Ethical approval was obtained from the Swedish Ethical Review Authority, Dnr 2023-06246-01 on January 30 2024. The study was registered at ClinicalTrials.gov with Identifier: NCT05973383 on 8 July 2023. Individuals who sign up for EviBody^®^ will be informed about the research study and invited to provide consent for data collection in the app. Controls will receive information about the study, via email, when registering interest and consent will be collected in the survey tool before they start to answer the questionnaire. The main risks of the study are related to data security and confidentiality. All data will be saved on a local server and study participants will be informed about the measures taken to secure confidentiality and trustworthiness. Personal data will be stored in a double-encrypted, three-level categorisation system, secured by pseudonymisation and by separating personally identifiable information from personally sensitive information. Authorized researchers can directly download prespecified and pseudonymised data from the system by identifying themselves with electronic identification and can only access data relevant to research purposes. The data will be stored for 15 years.

## Discussion

This study protocol aims to describe and provide details for a quasi-experimental study that evaluates the uptake and effectiveness of a digital intervention promoting healthy lifestyle behaviours. The study is novel in several aspects; 1) it will examine the effectiveness on well-being of three levels of a behaviour change intervention that takes place in a real-world setting, 2) a process evaluation is included to explore and descibe the circumstances of the intervention, 3) the study explores if the intervention and the specific intervention components vary in uptake among different population groups, and 4) the study is designed to evaluate programme theory by exploring mediators for change. Hence, this study aims to assist in translating research-derived strategies to real-world practice, evaluate scalable strategies, and pay attention to contextual circumstances and variations among population subgroups [58].

Evibody^®^ was developed to empower individuals to be in charge of their behaviour change process and take small steps forward based on their preferences and circumstances. It has a holistic and balanced focus on health rather than a focus on disease prevention or management. Therefore, well-being was chosen as the primary outcome. Well-being is defined as a resource for daily life embracing quality of life and the ability of people to contribute to the world with a sense of meaning and purpose [59]. As such, well-being is a construct that goes beyond disease diagnosis or having healthy lifestyle behaviours and takes a more holistic approach to health.

### Limitations and challenges

The study design has limitations, including the non-randomised allocation to intervention arms. Intervention participants intentionally register to use the app, and choose membership level. Intended app use will most probably vary among users and may as well change over time. However, intended app use will not be evaluated in this study, although it is of importance to research [23]. In addition, recruitment to the control group and reaching individuals comparable to the intervention participants may be challenging. These aspects present biases and caution should be made when interpreting the effectiveness of the study. We can not rule out that study participants report high levels of well-being at baseline introducing a ceiling effect and affecting the study’s ability to study effectiveness. In the conducted feasibility study, to which we recruited participants from the general adult population, the average rate of well-being was 58 and we expect similar rates in this study. Also, by excluding participants with well-being scores ≥ 70 from the analyses the ceiling effect is being considered.

There is a membership fee to the app, which makes it more accessible to high-income groups, who are more likely to pay out-of-pocket or have access to the app through benefits platforms. It is also probable that already health-engaged people with a high health literacy consent to participate in this research study to a higher degree than people with low health literacy. Failure to include people with sociodemographic variations will influence the ability to study all planned predicting factors for behaviour change, and will impact the external validity of the findings. We may decide to include more participants to be able to stratify analyses based on sociodemographics.

Another challenge is that with a dynamic app the intervention content and dose will change over time, between and within individuals. For example, the digital intervention will be further developed to adapt and optimise the behaviour change support based on user interactions, user feedback, and input from health coaches. Further, updates will be made according to trends in society and when new guidelines (e.g., dietary guidelines, work-related guidelines or healthcare-based guidelines) are released. To achieve traceability with the development process, new software versions will be assigned version numbers (e.g., version 1.12), and will be described carefully including detailed information about the adjustments made and the reason for the adjustments.

### Connected research

In addition to the studies mentioned in this paper, feasibility and optimisation studies are planned. The intention is to further enhance and adapt the intervention for different population groups through participatory research, including clinical populations (e.g., recovery from heart disease and cancer), groups of various socioeconomic statuses, and a variety professions (e.g., assistant nurses, construction workers, and factory workers).

## Conclusion

This study extends beyond assessing the effectiveness of a digital intervention. It delves into understanding which approaches are effective, for whom they are effective, and the specific conditions under which they are most beneficial.

## Supporting information

S1 File SPIRIT-Checklist. The SPIRIT-checklist listing page number with required information.
(DOCX)

S2 File Intervention components and behaviour change techniques. Description of intervention components and incorporated behaviour change techniques.
(DOCX)

S3 File Theoretical models for the planned prediction moderation and mediation analyses. (DOCX)

S4 File Study protocol approved by ethics commitee- English. (PDF)

S5 File Study protocol approved by ethics commitee- Swedish. (PDF)

S1 Video Intervention components. In the video the intervention components are described, and the layout and navigation of EviBody^®^ are shown.
(MOV)
