# Supplementary material for: Effectiveness and theory-based evaluation of a personalised digital intervention (EviBody®) for healthy and sustained lifestyle behaviours and well-being among adults: Study protocol for a real-world quasi-experimental study
Source: PLoS One. 2025 Oct 7;20(10):e0333201. doi: 10.1371/journal.pone.0333201 (PMC12503243; doi:10.1371/journal.pone.0333201)
Supplement: S2 File — Description of intervention components and incorporated behaviour change techniques. (DOCX) [file pone.0333201.s002.docx]

| **Intervention components** | **Behaviour change techniques** |
| --- | --- |
| **Onboarding and tutorials**  Pre-login onboardings for motivation and preparation, and post-login tutorials on functions in the service. | ***1. Goals and planning***  1.9 Commitment  ***5. Natural consequences***  5.1 Information about health consequences  ***7. Associations***  7.1 Prompts/cues  ***9. Comparison of outcomes***  9.1 Credible source  9.3 Comparative imagining of future outcomes  ***12. Antecedents***  12.5 Adding objects to the environment |
| **Individual action plan**  Screening questions, self-reflection, goal setting, action planning, tracking of behaviours and health outcomes. | ***1. Goals and planning*** 1.1 Goal setting (behaviour), 1.2 Problem solving, 1.3 Goal setting (outcome) 1.4 Action planning, 1.5 Review behaviour goals, 1.6 Discrepancy between current behaviour and goal, 1.7 Review outcome goals, 1.8 Behavioural contract, 1.9 Commitment,  ***2. Feedback and monitoring*** 2.2 Feedback on behaviour, 2.3 Self-monitoring of behaviour, 2.4 Self-monitoring of outcome(s) of behaviour, 2.7 Feedback on outcome(s) of behaviour,  ***4. Shaping knowledge*** 4.1 Instruction on how to perform a behaviour  ***5. Natural consequences*** 5.4 Monitoring of emotional consequences  ***6. Comparison of behaviour*** 6.1 Demonstration of the behaviour  ***8. Repetition and substitution***  8.1 Behavioural practice/rehearsal 8.2 Behaviour substitution, 8.4 Habit reversal, 8.7 Graded tasks  ***9. Comparison of outcomes*** 9.3 Comparative imagining of future outcomes |
| **Interaction and feedback**  Feedback on behaviours, digital rewards and customised push notifications, reminders, and prompts. | ***1. Goals and planning***  1.5 Review behaviour goals, 1.6 Discrepancy between current behaviour and goal, 1.7 Review outcome goals  ***2. Feedback and monitoring***  2.2 Feedback on behaviour, 2.7 Feedback on outcome(s) of behaviour  ***6. Comparison of behaviour***  6.2 Social comparison  ***7. Associations***  7.1 Prompts/cues,  7.3 Reduce prompts/cues  ***10. Reward and threat***  10.4 Social reward  ***11. Regulation***  11.3 Conserving mental resources  ***14. Scheduled consequences***  14.1 Anticipation of future rewards, 14.5 Rewarding completion,  ***15. Self-belief***  15.1 Verbal persuasion about capability |
| **Knowledge and inspiration**  A library containing educational content on health and behaviour change, exercise instruction videos, food recipes (including a link for web-based food purchasing), cooking instruction videos, meditation instruction videos, and relaxing music. Webinars and short articles with information and inspiration.  Prompts with suggestions personalised to the users' capability level and readiness for change. | ***4. Shaping knowledge***  4.1 Instruction on how to perform a behaviour, 4.2 Information about antecedents  ***5. Natural consequences***  5.1 Information about health consequences, 5.6 Information about emotional consequences  ***6. Comparison of behaviour***  6.1 Demonstration of the behaviour  ***8. Repetition and substitution***  8.1 Behavioural practice/rehearsal, 8.2 Behaviour substitution, 8.3 Habit formation  ***9. Comparison of outcomes***  9.1 Credible source  ***11. Regulation***  11.2 Reduce negative emotions, 11.3 Conserving mental resources  ***12. Antecedents***  12.1 Restructuring the physical environment, 12.2 Restructuring the social environment, 12.3 Avoidance/reducing exposure to cues for the behaviour, 12.4 Distraction, 12.6 Body changes  ***13. Identity***  13.1 Identification as role model  ***15. Self-belief***  15.1 Verbal persuasion about capability, 15.3 Focus on past success |
| **AI-Chat**  AI chat with digital coaching for an optimised action plan and realistic goal setting. | ***1. Goals and planning***  1.1 Goal setting (behaviour), 1.2 Problem solving, 1.3 Goal setting (outcome), 1.4 Action planning, 1.5 Review behaviour goals, 1.6 Discrepancy between current behaviour and goal, 1.7 Review outcome goals  ***4. Shaping knowledge***  4.2 Information about antecedents  ***8. Repetition and substitution***  8.2 Behaviour substitution, 8.4 Habit reversal, 8.7 Graded tasks  ***9. Comparison of outcomes***  9.1 Credible source, 9.2 Pros and cons, 9.3 Comparative imagining of future outcomes  ***12. Antecedents***  12.1 Restructuring the physical environment, 12.2 Restructuring the social environment, 12.3 Avoidance/reducing exposure to cues for the behavior,  12.4 Distraction  ***13. Identity***  13.2 Framing/reframing  ***15. Self-belief***  15.1 Verbal persuasion about capability, 15.3 Focus on past success,  15.1 Verbal persuasion about capability |
| **Community**  A community for social support, education, and inspiration. Including theme groups with multiple possibilities to interact, support and communicate. | ***1. Goals and planning***  1.2 Problem solving  ***3. Social support***  3.1 Social support (unspecified), 3.2 Social support (practical), 3.3 Social support (emotional)  ***5. Natural consequences***  5.1 Information about health consequences, 5.6 Information about emotional consequences  ***6. Comparison of behaviour***  6.1 Demonstration of the behaviour, 6.2 Social comparison, 6.3 Information about others’ approval  ***8. Repetition and substitution***  8.1 Behavioural practice/rehearsal, 8.2 Behaviour substitution  ***9. Comparison of outcomes***  9.1 Credible source  ***10. Reward and threat***  10.4 Social reward  ***11. Regulation***  11.3 Conserving mental resources  ***12. Antecedents***  12.1 Restructuring the physical environment, 12.2 Restructuring the social environment, 12.6 Body changes  ***13. Identity***  13.1 Identification as role model, 13.5 Identity associated with changed behaviour  ***15. Self-belief***  15.1 Verbal persuasion about capability, 15.3 Focus on past success |
| **Group coaching**  *With standard and premium memberships*  Standard and premium memberships include group coaching led by trained health coaches. These comprise digital live events and physical meetups with personal and interactive discussions and skill training. | ***1. Goals and planning***  1.2 Problem solving  ***3. Social support***  3.1 Social support (unspecified), 3.2 Social support (practical), 3.3 Social support (emotional)  ***5. Natural consequences***  5.1 Information about health consequences, 5.6 Information about emotional consequences  ***6. Comparison of behaviour***  6.1 Demonstration of the behaviour, 6.2 Social comparison, 6.3 Information about others’ approval  **7. Associations** 7.1 Prompts/cues  ***8. Repetition and substitution***  8.1 Behavioural practice/rehearsal, 8.2 Behaviour substitution  ***9. Comparison of outcomes***  9.1 Credible source  ***10. Reward and threat***  10.4 Social reward  ***11. Regulation***  11.2 Reduce negative emotions, 11.3 Conserving mental resources  ***12. Antecedents***  12.1 Restructuring the physical environment, 12.2 Restructuring the social environment, 12.6 Body changes  ***13. Identity***  13.1 Identification as role model, 13.5 Identity associated with changed behaviour  ***15. Self-belief***  15.1 Verbal persuasion about capability, 15.3 Focus on past success |
| **Individual coaching**  *Only with premium membership*  Premium membership includes individual digital consultations with trained health coaches based on motivational interviewing to support the behaviour change process and discuss the individual action plan. Before a consultation, the user answers some questions about current goals and well-being. | ***1. Goals and planning***  1.1 Goal setting (behaviour), 1.2 Problem solving, 1.3 Goal setting (outcome), 1.4 Action planning, 1.5 Review behaviour goals, 1.6 Discrepancy between current behaviour and goal, 1.8 Behavioural contract  ***2. Feedback and monitoring***  2.2 Feedback on behaviour, 2.3 Feedback on outcome(s) of behaviour  ***3. Social support***  3.1 Social support (unspecified), 3.2 Social support (practical), 3.3 Social support (emotional)  ***8. Repetition and substitution***  8.2 Behaviour substitution, 8.3 Habit formation, 8.4 Habit reversal, 8.7 Graded tasks  ***9. Comparison of outcomes***  9.1 Credible source, 9.2 Pros and cons  ***10. Reward and threat***  10.4 Social reward  ***14. Scheduled consequences***  14.1 Anticipation of future rewards  ***15. Self-belief***  15.1 Verbal persuasion about capability, 15.3 Focus on past success |
