# Supplementary material for: Effectiveness and theory-based evaluation of a personalised digital intervention (EviBody®) for healthy and sustained lifestyle behaviours and well-being among adults: Study protocol for a real-world quasi-experimental study
Source: PLoS One. 2025 Oct 7;20(10):e0333201. doi: 10.1371/journal.pone.0333201 (PMC12503243; doi:10.1371/journal.pone.0333201)
Supplement: S3 File — (DOCX) [file pone.0333201.s003.docx]

Theoretical models for the planned prediction, moderation and mediation analyses.

1. **Exploring factors predicting intervention outcomes**

**Aim**
Objective 3.1 To explore whether sociodemographic factors serve as predictors of intervention efficacy over a 24-month period.

**Statistical analysis**To test how belonging to various sociodemographic factors influence the outcomes of the digital intervention over time a predictive SEM model (latent Growth Model) will be used. Variables to be used are shown in table 1.

Sociodemographic factors   Growth trajectory of intervention outcomes over time

Figure 1. Theoretical model showing the relationship between sociodemographic factors as predictors and outcomes.

Table 1. Variables to be studied to explore factors predicting intervention outcome.

| **Sociodemographic groups** | **Engagement** | **Intervention outcomes** |
| --- | --- | --- |
| Age  Gender Occupation  Profession Education level  Birth country  Living area | Have registered goals and activities in the app (at least once per six month). | Achieved self-identified goals  Eating and physical activity habits  Mental health  Well-being |

1. **Exploring factors moderating intervention outcomes**

**Aim**
Objective 3.2 To explore whether sociodemographic factors serve as moderators of intervention efficacy over a 24-month period.

**Statistical analysis**
Multi-Group SEM will be used to test if sociodemographic factors act as moderators, influencing the strength or direction of the relationship between engagement and intervention outcomes. Variables to be used are shown in table 2.

1. Engagement → Intervention outcomes
(Does engagement increase intervention outcomes?)

2. Testing whether the structural paths differ across groups (e.g., younger vs. older participants, males vs. females)

Sociodemographic groups (e.g younger vs. older)

Engagement    Intervention outcomes

Figure 2. Theoretical model to test how belonging to various sociodemographic groups moderate the relationship between engagement with the app and outcomes.

Table 2. Variables to be studied to explore factors moderating intervention outcome.

| **Sociodemographic groups** | **Engagement** | **Intervention outcomes** |
| --- | --- | --- |
| Younger vs. older  Male vs female Working vs retired/on sick leave/unemployed Blue collar vs White collar  High vs low education  Birth country Sweden vs European vs non-European High income vs low Socioeconomically advantaged vs disadvantaged living area | Have registered goals and activities in the app (at least once per six month). | Achieved self-identified goals  Eating and physical activity habits  Mental health  Well-being |

1. **Exploring factors mediating intervention outcomes**

Objective 3.3 To examine whether engagement, motivation, self-efficacy, and perceived barriers mediate the associations between sociodemographic characteristics and changes in self-identified goals, eating and physical activity habits, mental health, and well-being over a 24-month period.

Model Structure

1. Path A:
   Engagement → Mediators
   (Does engagement increase motivation/self-efficacy/perceived barriers?)
2. Path B:
   Motivation/Self-efficacy/Perceived barriers → Outcome
   (Does motivation/self-efficacy/perceived barriers improve intervention outcomes?)
3. Path C (direct effect):
   Engagement → Outcome
   (Is there a direct effect of engagement on outcome, beyond motivation, self-efficacy and perceived barriers?)
4. Indirect effect:
   Engagement → Motivation, Self-efficacy and Perceived barriers→ Outcome
   (Is there a mediation pathway?)


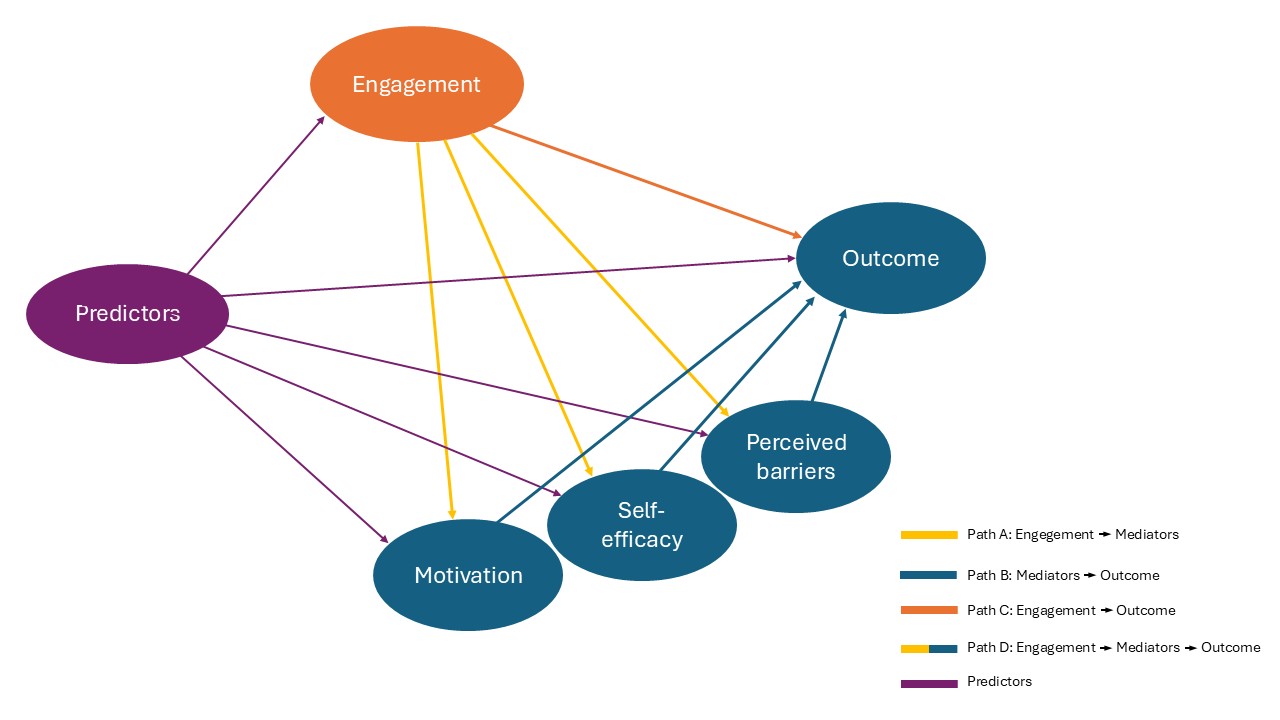


Figure 3. Theoretical model showing the hypothesised relationships between predictors, engagement, mediators and outcomes.

Table 3. Variables to be studied to explore factors mediating intervention outcome.

| **Engagement** | **Mediating factors** | **Intervention outcomes** | **Predictors** |
| --- | --- | --- | --- |
| Have regularly registered goals and activities in the app (at least once per six month). | Motivation  self-efficacy  Perceived barriers | Achieved self-identified goals  Eating and physical activity habits  Mental health  Well-being | Factors shown to be predictors in analysis 1. |
