# Supplementary material for: Effectiveness and theory-based evaluation of a personalised digital intervention (EviBody®) for healthy and sustained lifestyle behaviours and well-being among adults: Study protocol for a real-world quasi-experimental study
Source: PLoS One. 2025 Oct 7;20(10):e0333201. doi: 10.1371/journal.pone.0333201 (PMC12503243; doi:10.1371/journal.pone.0333201)
Supplement: S4 File — (PDF) [file pone.0333201.s004.pdf]

1. General information

Title of the investigation

Evaluation of an individually tailored digital service to promote healthy and sustainable behaviors and well-being

1.1 Reference number

CIV-23-07-043416

1.2 Amendments from prior versions of the CIP

N/A

1.3 Abbreviations and acronyms

BCT- Behavior Change Technique
CRF- Case Report Form, a list of the variables that will be collected and saved for research purpose
IB- Investigator´s Brochure, a protocol for the investigators and health coaches
ISI- Insomnia severity Index
MARS- Mobile Application Rating Scale
OLBI- The Oldenburg Burnout Inventory
KEDS- The Karolinska Exhaustion Disorder Scale
SUS- System Usability Scale
S-GSE General Self-Efficacy scale
RCT- Randomized Controlled Trial
RTCQ- Readiness To Change Questionnaire
WHO-5- World Health Organisation Well-Being Index

1.4 Sponsor and funding

Sponsor

Jenny Rossen, PhD, Sophiahemmet Högskola,
Inst. För Hälsofrämjande vetenskap,
Lindstedtsvägen 8, 114 86 Stockholm
Jenny.rossen@shh.se,
+46 84062985

Funding

Diabetesfonden (Dnr DIA2021-666)
Sophiahemmet Ideell Förening
Mälardalsområdets forskarskola

Additional funding has been applied for from e.g. Forte, AFA Försäkring

1.5 Principal investigator, coordinating investigator and investigation site

Principal investigator

Jenny Rossen, Med Dr, Sophiahemmet University, Department of Health Promoting Science
Principal investigator and responsible for the overall coordination of the investigation.
Jenny holds a Ph.D. in the subject area support for physical activity and has experience in designing, implementing, and evaluating behavior change interventions both quantitatively and qualitatively.

Investigation site
LongLife Active AB
Linjalvägen 6B, 187 66 Täby

Investigators
Julia Bergevi, MSc Nutrition, Quality Manager LongLife Active AB. Coordinating investigator
Julia will coordinate the investigation on-site, assuring the monitoring process according to the CIP and Investigator Brochure (IB).

Linn Sjöbeck, CPO, LongLife Active AB
Linn will assure the monitoring process according to the CIP and (IB).

Gustav Berggren, CTO, LongLife Active AB
Gustav will assure data monitoring plan is followed and secure the data.

Unn-Britt Johansson, professor, Sophiahemmet University, Department of Health Promoting Science
Unn-Britt has extensive research experience in the subject of living with long-term illnesses and using technical tools for treatment and decision support. Furthermore, Unn-Britt has solid experience in using questionnaires and designing and evaluating randomized controlled trials.

Maria Hagströmer, professor, Karolinska Institutet, Department of Neurobiology, Care Sciences and Society

Maria has extensive experience in practical work of supporting healthy lifestyle habits in various population groups. She also has extensive research experience in lifestyle change and measurement methodology, mainly in physical activity, and in the evaluation of innovative methods to support behavior change.

Susanne Andermo, assistant professor, Karolinska Institutet, Department of Neurobiology, Care Sciences and Society
Susanne has educational and research experience in public health and care sciences, with a focus on health promotion and physical activity. She has experience in research with different methodological approaches, primarily qualitative and implementation research.

Philip von Rosen, associate professor, Karolinska Institutet, Department of Neurobiology, Care Sciences and Society
Philip has over 10 years of experience in supporting healthy lifestyle behaviour of patients. He has also experience from research with different methodological approaches and advanced data analysis methods, mainly in physical activity research. Philip will assure that data analysis plan is followed.

Lena Kallings, associate professor, Swedish School of Sport and Health Sciences, GIH.
Lena has vast experiences in different interventions studies to promote physical and mental health, as well as health behaviour as physical activity and reducing prolonged sitting. Including

development, research and implementation of the Swedish PAP (Fysisk aktivitet på recept, FaR) national and international.

Clinical expertise
Therese Anderbro, assistant professor and clinical psychologist, department of Psychology, Stockholm University

Therese has vast experience as a clinical psychologist in both primary care and in psychiatric care. She also has experience in research regarding mental health in diabetes and in research evaluating intervention aimed at promoting behavior change in type 1 diabetes.

1.6 Overall synopsis of the clinical investigation

Study design: Post-market observational clinical investigation with a control group (a quasi-experimental study). The project also includes implementation and longitudinal studies.

Inclusion criteria (intervention arms): Adults ≥18 years who sign up for the service LongLife Active using BankID for identification, and consent to the research study.

Inclusion criteria (controls): Adults ≥18 years.

- *Exclusion criteria (intervention arms)*: Individuals who are discharged from the app due to refracting the terms of the service will be excluded. Subjects scoring >70 on well-being or subjects who appear as friends, colleagues, or family with anyone in the research or owner group will be excluded from the primary analyses.
- *Exclusion criteria (controls)*: Age ≤ 17 years. Is currently using a digital product or any other kind of support for behavior change (e.g. Livsstilsverktyget, Health Integrator, Weight Watchers). Is found to be a user of LongLife Active. Subjects scoring >70 on well-being or subjects who appear as friends, colleagues, or family with anyone in the research or owner group

will be excluded from the primary analyses.

Number of subjects: Intervention: 5000, Controls: 200

Duration of the intervention: the subjects will be followed up to 24 months.

Follow-ups: 3, 6, 12, 18, and 24 months

Objectives: The aim of this project is to evaluate the effectiveness of an individually tailored digital service aimed to support healthy habits on well-being among the adult population compared to a control sample of the general population.
Secondary objectives are to describe the internalization of the digital service, to evaluate the impact of the digital service on reaching self-identified goals, behavior change, and mental health, and to explore predicting and mediating factors for responses.

Primary endpoint: Well-being at 6 months
Secondary (intermediate) endpoints: Achieving self-identified goals, dietary and physical activity habits, and mental balance.

2. Identification and description of the investigational device

2.1 Identification of the device

LongLife Active AB, 559267-4492 is a Swedish Life Science company based in Täby, Sweden. LongLife Active is the manufacturer of the digital health service LongLife Active® that is a mobile phone application (app) available to download at Google Play and App Store. The development of the investigational device runs in-house at a proprietary Swedish platform in collaboration with an interprofessional scientific advisory board. The scientific advisory board includes independent researchers in the fields of physical activity, nutrition, nursing, psychology, and computer science. Agreements on non-funded collaborations are signed between LongLife Active AB and each researcher. Supplement 2 lists the involved researchers.

LongLife Active® is a digital health service (mobile phone application) classified as a medical software device class I, following Regulation (EU) 2017/745 of the European Parliament and of the Council of 5 April 2017 on medical devices (1). LongLife Active® is managed and regulated following the Swedish standards Medical devices – Quality management systems – requirements for regulatory purposes (ISO 13485:2016), and Health software – Part 2: Health and wellness apps – Quality and reliability (ISO/TS 82304-2:2021).

The digital service is intended for adults (≥18 years) in need of behavioral support to adopt healthy habits and establish a healthy lifestyle. The digital service is independent of any other device. Hence, no external accessories are needed to enable the intended performance and purpose of the device.

The user can connect the digital service with digital activity trackers or an external mobile phone application for automatic registration of the user's physical activity level. All accessories accepted to be synced with the digital service have been regarded within the reported justification of classification and risk class of the medical device. No medical accessories are planned to be connected with the digital service.

The digital service will be further developed to adapt and optimize the behavior support based on user feedback, analytics on user patterns, and inputs from health coaches. Further, it will be updated whenever new guidelines are released e.g. dietary guidelines or work-related and healthcare-based guidelines. To achieve traceability in the development process, new software versions of the investigational device will be assigned version numbers (e.g. version 1.12), and described carefully including detailed information on the adjustments made and the motivation/reason for the adjustments. Study subjects will be informed and directed to upload the new version when published to ensure that all users have access to the latest version. Downloaded software versions during the clinical investigation will be traceable anonymously to all users. The Investigator's Brochure (IB) supplement 3, covers documents and processes that ensure traceability in the development process of the investigational device during the clinical investigation and is available for all parties involved. The Quality manager and CPO at LongLife Active is responsible for maintaining the processes and ensuring traceability during the clinical investigation.

2.2 Description of the device

The medical device to be investigated is a digital service aimed to support healthy habits and improve well-being among the general adult population. The digital service includes three core subjects: 1) a Healthy diet, 2) Physical activity, and 3) Mental balance.

The service includes tracking and self-monitoring of behaviors, goal setting, action planning, feedback and digital rewards, a database with food recipes, a database with exercises, inspiration and skills training webinars and articles, and an AI chat. Figure 1 illustrates the core functions of the service. The included functions and applied behavior change techniques (BCTs) are presented in supplement 4, *Overview functions and BCTs*. Users may sign up for three levels of memberships. The first level (Basic) includes the beforementioned functions, the second level (Standard) also includes a community for social support, and the third level of subscription (Premium) includes a community and the possibility for private health consultations. The number of private health consultations is 3 for a 3 months subscription, 4 for a 6 months subscription, and 6 for a 12 months subscription.

Figure 1. Print screens from the device showing the core intervention components.

The digital service is assisted by artificial intelligence to direct the support to the individual timely and based on each user's needs, motivation, preferences, experiences, opportunities, and capabilities. A literature review undertaken during the development phase showed users of digital services wish for interface design and reminders to be customizable, and that the service is integrated with other services (such as shopping lists) (2). These are aspects that have been regarded to keep engagement over time. With a straightforward design, pictures, and short video instructions the service is customized for people with low skills in the Swedish language or with low digital literacy. Hence, no training or previous experience in digital health services is needed before using

the device. An AI chat is included as a core function to offer individualized support and guidance concerning the user's action plan and goals. Phrases and constructed dialogues of the chat have been developed by health professionals with expertise and experience in health coaching and motivational interviewing.

Based on the risk assessment informative prompts are available in the service to inform the user about realistic goals and actions when adopting a healthy and sustainable lifestyle, and to guide users in need of further support from health professionals. Moreover, report functions are available to alert inappropriate posts in the digital community. Health coaches leading individual- and group counseling in digital video meetings are trained in how to use the investigational device during a mandatory introduction course before operating in the service for the proposed clinical investigation. An introduction course is produced by the Head of health coaching and Quality manager and covers elements of security measures based on the risk assessment. Informed consent, including a description of the data security measures taken is collected from the subject before using the service.

Online technical support is available for all users and health coaches.

The supplement 3 (IB) includes security measures based on the risk assessment and operative guidelines for LongLife Actives health coaches.

3. Justification for the design of the clinical investigation

3.1 Rationale for the study design

Digital technologies based on artificial intelligence can provide personalized support for healthy behaviors and empower individuals to actively participate and track their health progress (3-5). The evidence for positive short-term effects of digital technologies on lifestyle change and health outcomes in controlled settings is rigorous (6-11) and using self-monitoring and goal setting as fundamental tools in interventions for behavior change is powerfully advised (9, 10, 12). Well-being is an aspect of health related to the WHO definition of health and is included in sustainable development goal 3 of the Agenda 2030. To the best of our knowledge, the impact of digital technologies promoting healthy habits on *well-being* is not well-studied.
Digital counseling versus simpler interventions using self-monitoring alone has been reported to have an additional effect on behavior change (12). However, the evidence is limited and studies comparing simpler interventions (e.g. providing pedometers or lifestyle advice) with more complex interventions are needed to establish best practices, and for cost-evaluation purposes (6, 12).
When properly designed, digital technologies providing behavior change techniques timely have the potential to improve short-term adherence to healthy behaviors (14, 15). Though, research has until now focused on short-term controlled efficacy trials and up-scaled long-term (> 12 months) implementation studies are rare (10, 12). A major challenge of digital products is high rates of abandonment (13, 14) and factors influencing engagement with digital technology need to be understood and overcome (6, 14). Internalization and implementation factors such as reach and dose, as well as determinants and mediators of efficiency, are rarely researched (5-8, 11). Moreover, real-world trials are needed to establish if digital interventions are effective for behavior change outside the controlled research setting (15, 16).

This study is a real-world post-market observational clinical investigation with a control group aimed to evaluate the impact of a digital service in improving well-being by supporting healthy lifestyle habits. A four-armed control condition will allow comparison between a control group recruited among the general population, and three levels of intervention among subscribers. The levels of intervention are I) core functions include self-monitoring, goal setting, action planning, an AI chat and databases with recipes and exercises; II) the same core functions + social support; II) the core functions + social support + individual counselling by a professional health coach. Randomisation of subjects to the different allocation arms is not appropriate because the users or their employers will pay for the digital service and thus choose the level of support.

3.2 Prior studies and development of the medical device

3.2.1 Prior research

This project builds on the knowledge and experiences obtained from Sophia Step Study, a three-armed randomized controlled trial evaluating self-monitoring of physical activity and additional counselling as support for self-management of physical activity (17, 18). A process evaluation showed high adherence to the interventions at twelve months and that applying digital self-monitoring to support physical activity was feasible both with and without counselling support. No reverse effects were reported (19). The interventions were effective in increasing physical activity after six months, and in maintaining physical activity over two years (18). Though, the results showed a large individual variation, both in baseline physical activity patterns and in the level of change in physical activity. An interview study revealed that the participants were appreciative of the personalized approach of the program, receiving feedback on health outcomes and positive reinforcement (20). Participating in the research study led to a reported increase in awareness and motivation for physical activity, establishment of new routines, and a feeling of control over the own health (20). Some frustration was expressed concerning the reliability of the pedometer in measuring steps accurately (20). These above-mentioned findings point to the importance of an individual approach, targeting individual circumstances, needs, and preferences.

3.2.2 Formative evaluation

Prior to this clinical investigation, an intervention planning phase was conducted based on a framework for developing behavioral interventions (21) and a guide to developing and planning digital interventions (22). This formative evaluation phase included a systematic review of users' perceptions of e- and mHealth services promoting physical activity and healthy diets (2). In addition to the systematic review, two qualitative studies have been carried out. One study explored users' and health care professionals' perspectives on e-health and technology as a support for physical activity in diabetes self-management (23). The other study explored health care professionals' views on supporting physical activity in primary care (24). The findings from the systematic review and the two qualitative studies served as the basis for the development of the digital service to be evaluated (the app LongLife Active). Figure 2 shows an illustration of the development phases of the research project.

Figure 2. The three phases of the overall research project and parallel activities by the product developers. The first two research phases comprised the development of the digital service and the third phase includes evaluation of the product when on the market.

3.2.3 Development of the medical device
In 2021 the digital service was developed using a co-development approach including various stakeholders (21, 22). In total 9 workshops were conducted with researchers, product developers, designers, and health care professionals along with numerous short meetings in smaller groups in-between the workshops. Supplement 2 lists the involved stakeholders and figure 3 shows an overview of workshop themes and major decisions throughout the co-development phase. The COM-B system proposing capability, opportunity, and motivation as conditions for behavior served as a structural framework for the workshop discussions (25). The process of tailoring functions and features in the app was guided mainly by the Social Cognitive Theory (26) and the Transtheoretical Model of Change (27). After the development and prototype testing the Behavior Change Technique Taxonomy v1 (28, 29) was applied to label the functions that were finally included in the digital service. Thus, the use of behavior change techniques allows the included functions to be evaluated as mediators, compared to other studies, and included in meta-analyses. The included functions and behavior change techniques are described in supplement 4.

Figure 3. Main discussed contents of workshops with scientific advisory board during the development phase.

3.3. Pre-clinical testing

A pilot study evaluating the feasibility of the digital service LongLife Active was completed in August 2022. The pilot study aimed to optimize the service to become usable and acceptable based on users' opinions. The subjects tested the service for three months. Validated questions on usability and acceptability of various functions were integrated into the service (30, 31) and engagement with the digital service have been evaluated by analysing analytics. The subjects were encouraged to leave continuous comments and feedback on the app by texting or speaking through a think-aloud function. Individual interviews and focus group discussions were arranged at the end of the test period to collect viewpoints on the digital service from users, health coaches, and program developers. A manuscript is in progress.

A total of 105 people showed an interest in participation, of these 55 participants signed up to use the digital service. Five participants dropped out due to various reasons, leaving 50 active users throughout the 12-week testing period. Most participants were recruited via social media (94%), most were female, and the majority were between the ages of 50-64. Regarding engagement with the app functions, 25 of the participants actively set at least one goal for behavior change. Most popular topic was physical activity, followed by mental balance and diet. The average goal success for both daily and weekly goals was 55.1%. In all, 39 participants had at least one activity from week 1 in the intervention period registered. In terms of engagement on a weekly basis with regards to registering activities, as expected fewer activities were registered later on in the intervention period; the number of activities registered dropped consecutively after week 5. Just over 80 activities were registered in week 1, with an increase to around 140 activities and staying steady at this level in weeks 2-5. This number then dropped to 60 activities in week 9 and further dropped to 20 activities in week 12. The most common (median) number of activities registered was 3. Across the 12-week intervention 14 participants wrote comments in the community function and 20 participants had at least one coaching session.

The response rate of the initial questionnaire was 64%. On a question about the initial screening questions, 59% agreed that these questions were relevant to their lifestyle. Concerning assistance with goal setting, 31% found that the LongLife Active service was good/very good in helping them to concrete their goals and 22% experienced the service was bad/very bad in concreting their goals. The goal setting function has been improved fundamentally since. In all, 69% felt good/very good and 40% very good about leaving personal or sensitive information in the service, and only 3% (1 participant) felt bad about this.

The response rate of the questionnaire covering the perceived usability of the service collected by the System Usability Scale (SUS) at week 3 was 30%. The overall SUS score was 50.0, which is an OK rating, one that is slightly below the average (32). Acceptability was assessed by the Mobile Application Rating Scale (MARS) at the same time point. The score is out of 5 and indicate overall app quality. The overall MARS score was 3.27, which is considered to be acceptable (3). The mean scores for all the sections were: engagement = 2.97, functionality = 2.83, aesthetics = 3.29 and information quality = 4. No adverse events or dissatisfaction were reported.

The seemingly low response rates to the questionnaires and reduced engagement over time were expected. The findings from the pilot study, especially the individual interviews, have powerfully guided further development and optimization of the digital service and the design of the clinical evaluation.

Another pilot study including 20 individuals with type 2 diabetes who tested the device for 1 months was completed in April 2023. The participants gave constructive feedback on the usability and value of the device. Twelve of the participants were interviewed and their comments have been very useful for adapting and optimizing the device, especially the action plan and goal setting functions. A manuscript describing the findings is in progress.

4. Benefits and risks of the investigational device, clinical procedure, and clinical investigation

Risk management activities are planned based on ISO 14155:2020 and will be performed throughout the clinical investigation. A risk-benefit analysis is described below as well as in the supplement 3. The subjects are informed of risks and mitigation of risks upon consenting.

4.1 Anticipated clinical benefits

The primary expected clinical benefit of regular use of and engagement with the digital service is improved well-being, else, the anticipated benefits depend on what behavior the subject chose to focus on. Eating and physical activity habits, sleep, stress, and exhaustion symptoms will be assessed as secondary outcomes. Additional benefits for the individuals that are expected to follow the anticipated improved physical activity levels and eating habits, although not tracked during the investigation, are reduced depressive symptoms and reduced cardiometabolic risk factors (e.g. overweight and obesity, blood lipids, metabolic control, blood pressure).

4.2 Anticipated adverse device effects

Adverse effects of digital support for behavior change are not obvious, and none have been reported in the scientific literature as far as we are aware. A systematic review including 113 RCTs investigating healthy diet and physical activity counselling reporting on adverse effects found no evidence for greater harm in the intervention versus the control group (33). Nevertheless, there are some identified concerns associated with the use of digital services for behavior change. The main risks that concern end-users are related to data security and confidentiality, reliability of the device,

and trustworthiness of the manufacturer (2, 23). Demotivation, self-confidence, self- consciousness, factors interrupting tracking, and data inaccuracy are common reasons for discontinued use (14).

Additional risks are increased screen time, perceived stress and excessive exercise and eating behaviors, potentially leading to orthorexia, and eating disorders. Filling out forms about mental health status and lifestyle behaviors may give rise to anxiety and low self-esteem. Not reaching self-identified goals and lack of expected results may cause a feeling of failure and hopelessness. Chat groups may be used to harass other subjects and encouragement and feedback can be misunderstood by subjective interpretation. Ultimately, behavior change will improve clinical values (e.g. blood lipids, metabolic control, blood pressure, pain, and sleep). Individuals on medication would conceivably need to adjust the dose of medication. There is a risk for relapse in behavior change and to re-adjust the medication.

4.3 Risks associated with participation in the clinical investigation

Study subjects register for the digital service on the premises to use it as a consumer product, not to be involved in research. Upon registration a request to participate in the research study is shown in the app with information about the research study and obligations of participation (supplement 5a Forskningspersonsinformation). Study subjects consent to share their data for research purposes and will be asked to answer questions in the app that has been provided by researchers. This may cause stress and distraction to the user's intended use of the service for improved health and well-being, and increased concerns about private privacy as the data will be transferred to the researchers and used for research purposes.

Except for the before mentioned adverse device effect, no other risks have been identified for subjects in the clinical investigation.

4.4 Possible interactions with concomitant medical treatments as considered under the risk analysis.

The risk of interactions with eventually concomitant medications is low. The subjects will be recommended to keep in contact with their responsible clinician to adjust medications if necessary.

4.5 Steps that will be taken to control or mitigate the risks.

The risks of data insecurity will be handled cautiously, and the subjects will be informed about the measures taken to secure confidentiality and trustworthiness with an informed consent. The system is built to never share any personal data with any party, applying pseudonymization with anonymous ID numbers for all users. Personal data will be stored in a double-encrypted, three-level categorization system to ensure data security, and personal integrity by separating personally identifiable information from personally sensitive information. The system has strictly limited access to personal data with regulated, monitored, and logged functions that enable access to limited and anonymous data for authorized parties only. For example, authorized researchers and health coaches must identify themselves with electronic identification to access strictly limited and anonymous data, only relevant to research or health coaching purposes. Overall, the system complies with the current requirements for security and monitoring, which is ensured by regular security checks and penetration testing by an external independent party.

The risk of perceived stress, excessive behaviors, anxiety, a feeling of failure, hopelessness, and low self-esteem are mitigated through support by setting realistic and progressive goals, encouragement, information, and webinars about behavior change and healthy habits. Alerts and report functions are built into the system to alarm excessive habits and low well-being score. World Health Organization's well-being index (WHO-5) (34) will be sent out monthly to the user. When low well-being is reported (score 50 or below) recommendations to healthcare providers is presented to the user.

A requirement for working as a health coach is owning a higher-level education as a health professional including at least 7.5 ECTS in health counselling. A mandatory introduction course for all new health coaches is produced by the Head of health coaching and Quality manager and covers elements of mandatory security measures based on the risk assessment. Prior to the coach session, the coach gets data on the user's action plan covering well-being scores and details about the user's action plan. A protocol and a guide for how and where to refer individuals in need of health care have been produced by the quality manager.

To prevent bullying and harassment between users, LongLife Actives health coaches manage and monitor the community. Also, alert and report functions are built into the system for users and coaches to alarm discourage and inappropriate posts in the community.

Overall, risk management activities will systematically be performed following the ISO 14155 framework throughout the clinical investigation. The identified residual risks will regularly be monitored and analyzed to ensure sufficient security measures. A Monitoring plan has been developed which covers processes of all risk management activities and security measures based on the risk assessment including operative guidelines for LongLife Actives health coaches. The Quality manager is responsible for risk management and educates all stakeholders involved in risk management activities.

4.6 Rationale for the benefit-risk ratio.

Expected benefits of the medical device outweighs the potential risks. On the first hand, there are anticipated benefits on well-being and behavioral outcomes. In addition to these anticipated benefits, previous studies have reported further empowerment and health literacy benefits of digital

interventions in relation to self-management. Examples of benefits are improved decisions making skills concerning the own health, perceived facilitation of self-management, an understanding of self-monitoring readings, increased awareness, a sense of control over a condition, and improved motivation to continue self-management activities (35). In this study the subjects will set self-identified goals and decide for themselves of a weekly plan for how to reach the goals in their own pace. Suggestions, encouragement, inspiration, and feedback will be tailored to preferences and identified needs of each individual. These are factors that potentially lead to improved empowerment and health literacy.

4.7 Data monitoring committee

The Data monitoring committee consists of the sponsor (Jenny Rossen) and investigators (Julia Bergevi, Linn Sjöbeck and Gustav Berggren). Monthly meetings are arranged unless more often is needed.

5. Objectives and hypothesis of the clinical investigation

5.1 Main objective

I) The primary objective of this project is to evaluate the effectiveness of an individually tailored digital service aimed to support healthy habits among the adult population on well-being compared to a control sample of the general population.

5.2 Secondary objectives

II) To study internalization and implementation factors such as reach and dose of the medical device across 6 months.

III) To explore the impact of three different levels of digital support for healthy habits on reaching self-identified goals, dietary and physical activity habits, and mental health after 6 months.

IV) To explore determining response of sociodemographic factors in meeting the self-identified goal, dietary and physical activity habits, mental health, and well-being.

V) To evaluate the intended mediators (engagement with the service, improved motivation, self-efficacy, and reduced perceived barriers) of the medical device on achieving the self-identified goal, improved dietary and physical activity habits, mental health, and well-being.

VI) To explore what app functions and features mediate improvements of the medical device on behavior change, mental health, and well-being.

VII) To explore the engagement of using the digital service and adherence to behavior change across 24 months.

VIII) To explore the patterns of achieving the self-identified goal, change in motivation, dietary and physical activity habits, mental health, and well-being across 24 months.

5.3 Hypotheses

1. The mean well-being score at 6 months of intervention is ≥12 score points higher in the intervention groups compared to the control group.

2. The impact on well-being is slightly stronger in the groups with higher level of support (Standard and Premium) compared to the group with lower level of support (Basic).

3. The medical device improves the individually targeted dietary habits, physical activity, and mental health among users at 6 months intervention compared to start of intervention.

4. Motivation, self-efficacy and achieving the self-identified goal mediate the effects of using the digital service on dietary and physical activity habits, mental health, and well-being.

5. The engagement with the service levels of across 24 months intervention for the group with less support (Basic) but maintains in the two groups with higher level of support (Standard and Premium).

6. The intervention effects on behavior change and well-being are maintained over 24 months in the two groups with higher levels of support (Standard and Premium) compared with the group with less support (Basic).

6. Design of the clinical investigation

6.1 Investigational device/Description of exposure

The medical device to be investigated is the mobile phone application (app) LongLife Active®. The app includes three core topics: 1) Healthy diet, 2) Physical activity, and 3) Mental balance. Supplement 4 shows the included intervention components and behavior change techniques. The service includes functions such as tracking and self-monitoring behavior and health outcomes, goal setting, action planning, a database with food recipes, a database with exercises, a database with relaxation exercises, skills training articles and webinars, live events, adjustable push notifications and gamification (rewards). If the user prefers there are options to pay extra for further social support: a version including a community (chat groups and group coaching sessions, live events and physical meetup events), and a version including the community plus the possibility to have a private health coach. The app is assisted by artificial intelligence to direct the support to the individual timely and based on the needs, motivation, preferences, experiences, opportunities, and capabilities of each user. The user decides which functions to use.

The literature review undertaken during the formative phase showed participants wished for interface design and reminders to be customizable, and that the service should be integrated with other services (such as shopping lists). These are examples of aspects that have been regarded to keep engagement over time. With a straightforward design, illustrations, and short video instructions the service is customized for people with low skills in the Swedish language or with low digital and/or health literacy.

No other medical devices or medications are to be used.

6.2 Subjects

6.2.1 Recruitment

The medical device will be launched and promoted by the company LongLife Active AB as a real-world project. The ambition is to reach individuals in need of lifestyle change, with a special emphasis on hard-to-reach groups. Health care services, pharmacies, employers, benefit platforms (e.g. Benify and Epassi) and social media will be targeted. Employers may apply for the health promoting subsidy, Friskvårdsbidrag, when employees sign up for LongLife Active. Health care patients prescribed physical activity (Fysisk aktivitet på recept, FaR) from their care provider is allowed a reduced fee on the membership of LongLife Active®.

Individuals who sign up for the service from September 2023, or as soon as ethical approval is obtained, will be informed about the research study and invited to consent for data extraction. Controls will be recruited through social media at the same time frame.

Inclusion criteria (intervention arms):

- adults ≥18 years who sign up for the service LongLife Active using BankID for identification.
- consenting to the research study.

Inclusion criteria (controls):

- adults ≥18 years.

Exclusion criteria (intervention arms):

- individuals who are discharged from the app due to refracting the terms of the service
- subjects scoring >70 on well-being will be excluded from the primary analyses (36)
- subjects who appear as friends, colleagues or family with anyone in the research or owner group.

Exclusion criteria (controls):

- age ≤ 17 years
- is currently using a digital product or any other kind of support for behavior change (e.g. Livsstilsverktyget, Health Integrator, Weight Watchers).
- is found to be a user of LongLife Active.
- subjects who appear as friends, colleagues or family with anyone in the research or owner group
- subjects scoring >70 on well-being will be excluded from the primary analyses (36).

Sample size: The estimation is to reach 5000 consenting individuals in total for the intervention arms and 200 controls.

Total expected duration for each subject's participation: (intervention arms: 24 months, (controls)

Drop- outs and discontinued use of the digital service: Subjects who notify they would like to withdraw from the research study are disconnected to the data base. Subjects who discontinue using the digital service will be invited to continue the research participation and to answer the questionnaires online.

6.3 Procedures for data collection

6.3.1 Procedures

This investigation will take place outside the clinical practice. Data will be collected by self-reported instruments (questionnaires) and analytics from the digital service. An exception from self-reported measures is that an activity monitor will be used to measure physical activity and sedentary behavior besides self-reported instruments. Supplement 6 Case Report Form shows the included questionnaires with details on specific questions and response alternatives. Reminders to answer the questionnaires will be pushed out weekly for 4 weeks until the subject has answered (max 4 reminders). The activity monitor will be posted to the subjects together with instructions and a log to write the time when the monitor was put on and off during the day. It should be placed on the thigh and worn for 7 consecutive days. The subject will be asked to fill out the diary for wake-up time and bedtime, and for major physical activities performed during the day.

Answering questionnaires and wearing the activity monitor is voluntarily, and if the subject does not answer it will not affect his/her use of the app.

There is a risk of selection bias affecting the results. We believe highly motivated subjects (responders) possibly answer the questionnaires to a larger extent than subjects facing more challenges.

6.3.2 Data collection

Data will primarily be collected through the app as self-reported answers to questionnaires and as analytics. The Case Report Form in supplement 6 shows the questions and response alternatives for each instrument. Table 1 shows an overview of time points and instruments used for data collection.

Reach

Information on promotion channels and data on sales statistics will be collected from the provider. This data is not connected to the subjects. Demographics: age, gender, occupational status, profession, education, country of origin and living area, as well as a question on how the subject got information about the service will be collected by questions when signing up.

Fidelity and dose

User activity patterns across time will be aggregated per week and described for the core functions: *Individual action plan, Coaching, Community* and *Platform of knowledge and inspiration*. Adherence with app use will be estimated using the frequency of weekly use across the tracking period. Supplement 6 Case Report Form shows the variables to be collected for this purpose.

Adaptations

Qualitative data from the support service chat, and from continuous dialogues with health coaches, recipe providers and staff maintaining the digital service and records over notable adaptations and changes made to the service will continuously be collected. Details and routines are described in the IB (Supplement 4).

Usability

Perceived usability of the service will be collected by the System Usability Scale (SUS) (31) and acceptability by Mobile Application Rating Scale Instrument for Evidence-Based Evaluation (MARS), (30).

Mediating factors and mechanisms of impact
Motivation will be assessed by the readiness to change questionnaire (RTCQ) based on the transtheoretical model (37, 38). Self-efficacy will be evaluated by the General Self-Efficacy scale (S-GSE) adapted for behaviour change (39). Barriers to healthy behavior will be evaluated by the Barriers to being active quiz (CDC Road to health barriers) adapted for barriers in trying to eat healthier and to recover (40).

Outcome measures

Mental health
Well-being will be assessed by the WHO-5 Well-Being Index (34). The WHO-5 comprises the following five items: being cheerful and in good spirits; being calm and relaxed; feeling active and vigorous; feeling fresh and rested when waking up in the morning; and having an interest in day-to-day activities. Six response alternatives are scored from 5 (All of the time) to 0 (At no time). The total raw score which ranges from 0 to 25 is multiplied by 4 to calculate the final score. The final score range goes from 0 = worst imaginable well-being to 100 = the best imaginable well-being. Stress will be assessed by the Karolinska Exhaustion Disorder Scale, a self-rating scale for stress-induced exhaustion disorder (41). Burnout will be assessed by the Oldenburg Burnout Inventory (OLBI) (42). Sleep will be assessed by the self-reported number of hours of sleep per night and ISI – Insomnia Severity Index (43). Four questions have been developed specifically for this study to assess mental balance.

Goal achievement
Reaching the self-identified goal will be evaluated by collecting information on number of days/week the self-identified goal was met during the past month. Confidence for reaching the goal will also be asked for on a scale 1 low confidence to 6 high confidence.

Dietary habits
Dietary behaviours will be assessed by questions from the Social Board of Health and Welfare (44), with the addition of some study specific questions on plant based diet.

Physical activity and sedentary behaviors
Physical activity and sedentary behaviors will be collected by questions from the Social Board of Health and Welfare (44) with additional questions on active transport and physical activity at work. In a randomized sample of individuals having increased physical activity as a goal (n=300) physical activity will also be measured objectively by the use of the three-axial accelerometer-based device Fibion (Fibion Inc, Jyväskylä, Finland) (45) or a similar device. Daily number of steps will be obtained from app analytics.

Aspect	Variable	Instrument	Time point (month)						
			0	1	3	6	12	18	24
**Demographics**									
	Age	Year of birth	x						
	Gender	SCB	x						
	Occupation	No specific	x						
	Profession	No specific	x						
	Education	No specific	x						
	Living area	No specific	x						
	Country of origin	No specific	x						
**Well-being and mental health**									
	Well-being	WHO-5 Well-being index	x	x	x	x	x	x	x
	Stress	The Karolinska Exhaustion Disorder Scale (KEDS)	x		x	x	x	x	x
	Burnout	The Oldenburg Burnout Inventory (OLBI)	x		x	x	x	x	x
	Sleep	Self-reported number of hours at sleep	x		x	x	x	x	x
		Sleep diary*							
		ISI – Insomnia Severity Index	x		x	x	x	x	x
	Mental balance	No specific	x		x	x	x	x	x
**Goal achievement**									
	Confidence in reaching the goal	No specific	x		x	x	x	x	x
	Days/week the self-identified goal was met during the past month	Analytics	x	x	x	x	x	x	x
**Mediators**									
	Self-efficacy	The General Self-Efficacy scale (S-GSE)	x		x	x	x	x	x

	Motivation	Readiness to change questionnaire (RTCQ)	x		x	x	x	x	x
	Barriers	CDC Road to health barriers	x		x	x	x	x	x
**Lifestyle habits**									
	Physical activity	Daily number steps from the app**	x	x	x	x	x	x	x
		Daily minutes in different intensities and sedentary behavior	x			x	x	x	x
		Questions from the National Board of Health and Welfare on active transport and physical activity at work.	x			x	x	x	x
	Dietary habits	Questions from the National Board of Health and Welfare and additional questions on plant-based food.***	x			x	x	x	x
**Usability**									
Usability		System Usability Scale (SUS)		x	x				
Acceptability		Mobile Application Rating Scale (MARS)		x	x				
**Engagement**									
	Use of action plan	Analytics	x		x	x	x	x	x
	Attendance at community tutorials och coaching	Analytics	x		x	x	x	x	x
	Number visits at knowledge and inspiration databases	Analytics	x		x	x	x	x	x

*Applicable only to subjects who have chosen mental balance and sleep for behavior change
** Applicable only to subjects who have chosen physical activity for behavior change
*** Applicable only to subjects who have chosen diet for behavior change

6.4 Monitoring plan

In this project most of the procedures are built into the app and are made automatic. Questionnaires and reminders to answer the questionnaires will be pushed through the app and data will be retracted from the continuously saved analytics. Supplement 3 shows the Case Report Form with variables that will be extracted for each individual.

All data is pseudonymized and stored in a three-level categorized and double-encrypted server to ensure personal integrity and security. The research study is managed as a separate module of the system, which enables automatic and anonymous communication with the research subjects, and safe data transfer limited to the research purposes on an individual- and group level without sharing any personally identifiable information. Transfer of data from the server to the principal investigator will be made through a regulated and monitored web platform with obligatory electronic identification for access.

Time points for the specific questionnaires are shown in table 1. The time for pushing out questionnaires is programmed at start and controlled by the investigators.

Routines for documentation on adverse effects is described in the IB (supplement 3). All involved personnel: quality manager, health coaches, webinar hosts and else are introduced to the IB and trained in the documentation process.

The IB will be saved as an online file to make sure everyone has the latest version. Changes made to the IB are highlighted and logged at the last page. Before changes are made a version of the IB is saved, named with version number and date.

Parts of the monitoring reports will be published in scientific journals as a process evaluation describing implementation factors, reach and engagement.

7. Statistical design and analysis

7.1 Analysis population

For the endpoint well-being an intention-to-treat approach will be applied. By using mixed model for repeated measures all subjects with two or more measurements will be included in the analysis and manual imputation it not needed. For the intermediate outcomes dietary habits, physical activity, and mental health only subjects who have set up change goals within the area will be analysed.

7.2 Descriptive statistics

Figure 5 shows a logic model for the project with the anticipated predictors, mediators, intermediate outcomes, and endpoints. Descriptive statistics will be applied to report the reach of the intervention including demographics and user engagement patterns (analytics over time) for intervention dose. In addition, user engagement patterns will be specified for subgroups (age, gender, employment status, profession, educational level, residence in urban, rural, or semi-rural area and country of origin). The data will be examined for outliers, normality, and missing data. Mean and standard deviation will be given for normally distributed continuous variables, median and range for continuous variables with skewed distribution, and number and percentage for categorical and dichotomous values.

Change in mediators, intermediate outcomes and the endpoint well-being will be reported between baseline and each follow-up time point, presented by intervention group. Mean differences and standard deviations will be used for continuous variables and number and percentage change for categorical and dichotomous variables.

Figure 5. Logic model for the digital service LongLife Active showing the predicted underlying pathway and responses.

7.3 Analytical procedures

Change in the endpoint well-being (as total score) and the intermediate outcomes dietary habits, physical activity, and mental health, will be analysed across time and between groups using mixed model for repeated measures, and reported as estimates with confidence intervals. The model will include intervention level and time (0, 6, 12, 18 and 24 months).

To investigate correlations and associations of demographic factors and user engagement with behavior change, mental balance, and well-being, structural equational modelling and mixed models will be applied.

Regression analysis and mixed models will be applied to evaluate mediating factors and responsiveness. These analyses will explore if high levels of motivation, self-efficacy and reduced perceived barriers mediates improvements in reaching the self-identified goal, behavior, mental health, and well-being and whether improvements in motivation, behavior, and mental health mediate well-being. Cut off values for the intermediate outcomes will be derived based on baseline values and findings from similar studies.

The threshold for statistical significance will be set at $p < 0.05$.

Qualitative data (chat groups and dialogues with included actors) will be analyzed by content analysis (46).

7.4 Sample size calculation

The endpoint well-being will be assessed using the WHO-5 Well-being Index (34). In our pilot study the mean score at baseline was 55. The instrument has to our knowledge not been applied on a general Swedish population. In Danish general population surveys, the mean WHO-5 score was 60-70 (sd=19.0-19.9) (36, 47, 48). A change of 10-20 points in WHO-5 has been proposed as a clinically relevant change (36, 49). In up-scaled interventions 60% lower effects are expected (50). We therefore aim at a difference of 12 points in the score between intervention groups and control group as well as between baseline and 6 months. A fluctuation in well-being is considered normal (36), subjects with well-being score >70 are not expected to improve and will be excluded from the analysis. In the pilot study 21% of participants reached 70 points at baseline.

Desirable sample size was calculated to detect an effect of 12 points (36, 51) based on the assumption of two-tailed testing, an alpha error $\alpha=0.05$ and power $1-\beta=0.90$ and sd=19.9 (48). To detect a medium standardised effect size of 0.5 (Cohen's d) for the primary outcome well-being at 6 months a sample size of at least 25 subjects per group is recommended. Expected drop-out rate due to withdrawal and lost to follow-up at six months is 75 %. Based on the expected drop-out rates and on excluding 20% from the analysis due to baseline score >70, we need to recruit at least 500 subjects to assure 25 subjects in each intervention arm. In the control group we assume subjects to be excluded based on using a digital product for behavior change and strive to include 200 subjects.

The secondary outcomes are exploratory due to the person-centred design of the service. The subjects will set individual goals and possibly change goals during the intervention. Likewise, habits and behavior change are largely changeable due to circumstances. The current research is not immense enough to guide a decision for minimal expected effects when individual goals are set (e.g., reducing weekly servings of red meat). A larger sample size than what is required for the primary

endpoint well-being is desired. Similarly, the secondary analyses of mediating factors and responsiveness require a large sample size. We therefore aim to include 5000 subjects.

8. Data management

Data will be retracted from the repeatedly saved analytics. During the pilot study syntax/algorithms for data output were developed, tested, and refined. Several datasets will be constructed as Excel-files and merged to one single file (master dataset). Merging data (based on subject-ID) and resolving data queries will be made by two researchers in dialogue with the data manager from the investigator site (LongLife Active). Database cleaning, rectifying errors and resolving issues will be made by two researchers in collaboration with the investigators.

The research study is managed as a separate module of the system, which enables automatic and anonymous communication with the research subjects, and safe data transfer limited to the research purposes on an individual- and group level without sharing any personally identifiable information. Transfer of data from the server to the principal investigator will be made through a regulated and monitored web platform with obligatory electronic identification for access.

At Sophiahemmet University data will be stored at Sunet Drive for Sophiahemmet University.

9. Amendments to the CIP

The CIP is saved at OneDrive for Sophiahemmet University and the latest version is available to the investigators and the research group as a shared document. Amendments to the CIP are as made as comments in the document. When the CIP is updated, old versions will be saved, and the amendments will be logged in the old version as well as in a separate document (CIP log).

10. Deviations from the clinical investigation plan

Any deviations from the CIP will be documented in a log (a separate Word-file, CIP log) along with reasons to the deviations and date. When the CIP is updated, old versions will be saved.

11. Device accountability

Access to the investigated medical device will be controlled using electronic identification (BankID). Subjects in the control group will be excluded if they answer they are using some digital support for behavior change. In addition, a comparison of names and email addresses it will be made to check that the control subjects are not using the investigated medical device.

12. Statements of compliance

Etiska överväganden enligt Annex A12

13. Informed consent process

Individuals who sign up for the digital service LongLife Active from October 2023 (or as soon as ethical approval is obtained) will be informed about the research study (informed consent in supplement 5a) and invited to consent for data extraction in the app. Controls will be recruited through social media and trough large companies and will be sent the detailed information by email (supplement 5b). Control subjects consent in the survey tool before starting to answer the questionnaire.

14. Adverse events, adverse effects, and device deficiencies

Alerts and report functions are built into the system to alarm excessive habits and low well-being score. The quality manager and health coaches manage and monitor alerts and inappropriate posts in the community. A Monitoring plan has been developed which covers processes of all risk management activities and security measures based on the risk assessment including operative guidelines for LongLife Actives health coaches. The Quality manager is responsible for risk management and educates all stakeholders involved in risk management activities. Adverse events and issues that are arising are logged and discussed among the investigators and sponsor and are at first hand alleviated.

15. Suspension or premature termination of the clinical investigation

The intervention subjects are included in the research study and contributing with data until 25 months have passed since registration of the first activity, or until reporting a wish to withdraw. Control subjects are included in the research study and contributing with data until 7 months have passed since submitting the first questionnaire, or until reporting a wish to withdraw. Reasons to terminate the research study are if large quantities of complaints are made about the digital service or about the burden of answering questions, or if 5000 subjects are not reached within 5 years.

16. Publication policy

The research project will be part of a post doc project and integrated in two or more doctoral theses. Thus, the research findings will be disseminated through peer reviewed open access journals that are recognized and indexed in PubMed. The Vancover guidelines by ICMJE will be followed to decide authorship order and be transparent of author contributions. Supplement 7 shows the planned studies and authorships that has been agreed upon.
The scientific results will also be disseminated through publishing in popular science journals and presentations at national and international conferences and seminars. At Sophiahemmet researchers collaborate very close with the marketing department when communicating with the wider society. The channels och types of information will be adjusted and focused to the target groups. Here we present at broad plan for channels.

Dissemination to the population

The research group has established networks and channels for research dissemination to the population, e.g. newsletters and social media that will be used. The company providing the app will spread updated information through social media and newsletters. Users of the app will regularly be updated about the research progress through short articles, videos, and webinars at a page for the purpose within the app.

Dissemination to politicians and practitioners
The results will be presented in scientific journals, professional journals and networks, through social media and through presentations at national and international conferences.

17. Bibliography

1. Regulation (EU) 2017/745 of the European Parliament and of the Council of 5 April 2017 on medical devices, amending Directive 2001/83/EC, Regulation (EC) No 178/2002 and Regulation (EC) No 1223/2009 and repealing Council Directives 90/385/EEC and 93/42/EEC.
2. Bergevi J, Andermo S, Woldamanuel Y, Johansson UB, Hagströmer M, Rossen J. User Perceptions of eHealth and mHealth Services Promoting Physical Activity and Healthy Diets: Systematic Review. JMIR Hum Factors. 2022;9(2):e34278.
3. Global diffusion of eHealth: making universal health coverage achievable: report of the third global survey on eHealth. World Health Organization; 2017. Contract No.: 978-9-24-151178-0.

4. Phillips SM, Cadmus-Bertram L, Rosenberg D, Buman MP, Lynch BM. Wearable Technology and Physical Activity in Chronic Disease: Opportunities and Challenges. American Journal of Preventive Medicine. 2018;54(1):144-50.
5. Fiedler J, Eckert T, Wunsch K, Woll A. Key facets to build up eHealth and mHealth interventions to enhance physical activity, sedentary behavior and nutrition in healthy subjects – an umbrella review. BMC Public Health. 2020;20(1):1605.
6. Hutchesson MJ, Gough C, Müller AM, Short CE, Whatnall MC, Ahmed M, et al. eHealth interventions targeting nutrition, physical activity, sedentary behavior, or obesity in adults: A scoping review of systematic reviews. Obesity Reviews. 2021;22(10):e13295.
7. Robert C, Erdt M, Lee J, Cao Y, Naharudin NB, Theng YL. Effectiveness of eHealth Nutritional Interventions for Middle-Aged and Older Adults: Systematic Review and Meta-analysis. Journal of medical Internet research. 2021;23(5):e15649.
8. Romeo A, Edney S, Plotnikoff R, Curtis R, Ryan J, Sanders I, et al. Can Smartphone Apps Increase Physical Activity? Systematic Review and Meta-Analysis. Journal of medical Internet research. 2019;21(3):e12053.
9. Kwan RYC, Salihu D, Lee PH, Tse M, Cheung DSK, Roopsawang I, et al. The effect of e-health interventions promoting physical activity in older people: a systematic review and meta-analysis. European Review of Aging and Physical Activity. 2020;17(1):7.
10. Ferguson T, Olds T, Curtis R, Blake H, Crozier AJ, Dankiw K, et al. Effectiveness of wearable activity trackers to increase physical activity and improve health: a systematic review of systematic reviews and meta-analyses. The Lancet Digital Health. 2022;4(8):e615-e26.
11. Schoeppe S, Alley S, Van Lippevelde W, Bray NA, Williams SL, Duncan MJ, et al. Efficacy of interventions that use apps to improve diet, physical activity and sedentary behaviour: a systematic review. International Journal of Behavioral Nutrition and Physical Activity. 2016;13(1):127.
12. Vetrovsky T, Borowiec A, Juřík R, Wahlich C, Śmigielski W, Steffl M, et al. Do physical activity interventions combining self-monitoring with other components provide an additional benefit compared with self-monitoring alone? A systematic review and meta-analysis. British journal of sports medicine. 2022:bjsports-2021-105198.
13. Alvarado MM, Kum HC, Gonzalez Coronado K, Foster MJ, Ortega P, Lawley MA. Barriers to Remote Health Interventions for Type 2 Diabetes: A Systematic Review and Proposed Classification Scheme. Journal of medical Internet research. 2017;19(2):e28.
14. Attig C, Franke T. Abandonment of personal quantification: A review and empirical study investigating reasons for wearable activity tracking attrition. Computers in Human Behavior. 2020;102:223-37.
15. Vandelanotte C, Duncan MJ, Kolt GS, Caperchione CM, Savage TN, Van Itallie A, et al. More real-world trials are needed to establish if web-based physical activity interventions are effective. British journal of sports medicine. 2019;53(24):1553-4.
16. Ogilvie D, Adams J, Bauman A, Gregg EW, Panter J, Siegel KR, et al. Using natural experimental studies to guide public health action: turning the evidence-based medicine paradigm on its head. J Epidemiol Community Health. 2020;74(2):203-8.

17. Rossen J, Yngve A, Hagströmer M, Brismar K, Ainsworth BE, Iskull C, et al. Physical activity promotion in the primary care setting in pre- and type 2 diabetes - the Sophia step study, an RCT. BMC Public Health. 2015;15:647.
18. Rossen J, Larsson K, Hagströmer M, Yngve A, Brismar K, Ainsworth B, et al. Effects of a three-armed randomised controlled trial using self-monitoring of daily steps with and without counselling in prediabetes and type 2 diabetes—the Sophia Step Study. International Journal of Behavioral Nutrition and Physical Activity. 2021;18(1):121.
19. Rossen J, Hagströmer M, Yngve A, Brismar K, Ainsworth B, Johansson U-B. Process evaluation of the Sophia Step Study- a primary care based three-armed randomized controlled trial using self-monitoring of steps with and without counseling in prediabetes and type 2 diabetes. BMC Public Health. 2021;21(1):1191.
20. Rossen J, Lööf H, Yngve A, Hagströmer M, Brismar K, Johansson U-B. 'This is why I'm doing a lot of exercise' — a qualitative study of participant's experiences of the Sophia Step Study. International Diabetes Nursing. 2017;14(2-3):99-104.
21. Morrison LG, Muller I, Yardley L, Bradbury K. The person-based approach to planning, optimising, evaluating and implementing behavioural health interventions. The European health psychologist. 2018;20:464-9.
22. West R, Michie S. A guide to development and evaluation of digital behaviour interventions in healthcare. London: Silverback Publishing; 2016.
23. Woldamanuel Y, Rossen J, Andermo S, Bergman P, Åberg L, Hagströmer M, et al. Perspectives on Promoting Physical Activity Using eHealth in Primary Care by Health Care Professionals and Individuals With Prediabetes and Type 2 Diabetes: Qualitative Study. JMIR Diabetes. 2023;8:e39474.
24. Larsson K, Hagströmer M, Rossen J, Johansson U-B, Norman Å. Health care professionals' experiences of supporting persons with metabolic risk factors to increase their physical activity level – a qualitative study in primary care. Scandinavian Journal of Primary Health Care. 2023:1-16.
25. Michie S, van Stralen MM, West R. The behaviour change wheel: A new method for characterising and designing behaviour change interventions. Implementation Science. 2011;6(1):42.
26. Bandura A. Social foundations of thought and action : a social cognitive theory. Englewood Cliffs, N.J.: Prentice-Hall; 1986.
27. Prochaska JO, DiClemente CC, Norcross JC. In search of how people change. Applications to addictive behaviors. The American psychologist. 1992;47(9):1102-14.
28. Michie S, Richardson M, Johnston M, Abraham C, Francis J, Hardeman W, et al. The behavior change technique taxonomy (v1) of 93 hierarchically clustered techniques: building an international consensus for the reporting of behavior change interventions. Annals of behavioral medicine : a publication of the Society of Behavioral Medicine. 2013;46(1):81-95.
29. Corker E, Marques M, Johnston M, West R, Hastings J, Michie S. Behaviour change techniques taxonomy v1: Feedback to inform the development of an ontology [version 2; peer review: 1 approved, 1 approved with reservations]. Wellcome Open Research. 2023;7(211).
30. Stoyanov SR, Hides L, Kavanagh DJ, Zelenko O, Tjondronegoro D, Mani M. Mobile app rating scale: a new tool for assessing the quality of health mobile apps. JMIR mHealth and uHealth. 2015;3(1):e27-e.
31. Lewis JR. The System Usability Scale: Past, Present, and Future. International Journal of Human–Computer Interaction. 2018;34(7):577-90.
32. Bangor A, Kortum PT, Miller JT. Determining what individual SUS scores mean: adding an adjective rating scale. Journal of Usability Studies archive. 2009;4:114-23.
33. Patnode CD, Redmond N, Iacocca MO, Henninger M. Behavioral Counseling Interventions to Promote a Healthy Diet and Physical Activity for Cardiovascular Disease Prevention in Adults Without Known Cardiovascular Disease Risk Factors: Updated Evidence Report and Systematic Review for the US Preventive Services Task Force. Jama. 2022;328(4):375-88.
34. Topp CW, Østergaard SD, Søndergaard S, Bech P. The WHO-5 Well-Being Index: A Systematic Review of the Literature. Psychotherapy and Psychosomatics. 2015;84(3):167-76.

35. Morton K, Dennison L, May C, Murray E, Little P, McManus RJ, et al. Using digital interventions for self-management of chronic physical health conditions: A meta-ethnography review of published studies. Patient education and counseling. 2017;100(4):616-35.
36. Bech P, Lindberg L, Moeller SB. The Reliable Change Index (RCI) of the WHO-5 in primary prevention of mental disorders. A measurement-based pilot study in positive psychiatry. Nordic Journal of Psychiatry. 2018;72(6):404-8.
37. Bock BC, Marcus BH, Rossi JS, Redding CA. Motivational readiness for change: Diet, exercise, and smoking. American Journal of Health Behavior. 1998;22(4):248-58.
38. Forsberg L, Ekman S, Halldin J, Rönnberg S. The readiness to change questionnaire: reliability and validity of a Swedish version and a comparison of scoring methods. Br J Health Psychol. 2004;9(Pt 3):335-46.
39. Löve J, Moore CD, Hensing G. Validation of the Swedish translation of the General Self-Efficacy scale. Qual Life Res. 2012;21(7):1249-53.
40. Kearney JM, McElhone S. Perceived barriers in trying to eat healthier--results of a pan-EU consumer attitudinal survey. Br J Nutr. 1999;81 Suppl 2:S133-7.
41. Besèr A, Sorjonen K, Wahlberg K, Peterson U, Nygren A, Asberg M. Construction and evaluation of a self rating scale for stress-induced exhaustion disorder, the Karolinska Exhaustion Disorder Scale. Scandinavian journal of psychology. 2014;55(1):72-82.
42. Demerouti E, Bakker AB, Vardakou I, Kantas A. The convergent validity of two burnout instruments: A multitrait-multimethod analysis. European Journal of Psychological Assessment. 2003;19(1):12.
43. Bastien CH, Vallières A, Morin CM. Validation of the Insomnia Severity Index as an outcome measure for insomnia research. Sleep Med. 2001;2(4):297-307.
44. Socialstyrelsens frågor om levnadsvanor: Socialstyrelsen; [Available from: https://www.socialstyrelsen.se/globalassets/sharepoint-dokument/dokument-webb/nationella-riktlinjer/levnadsvanor-fragor-om-levnadsvanor.pdf.
45. Yang Y, Schumann M, Le S, Cheng S. Reliability and validity of a new accelerometer-based device for detecting physical activities and energy expenditure. PeerJ. 2018;6:e5775-e.
46. Krippendorff K. Content analysis : an introduction to its methodology. Thousand Oaks, Calif. ;: SAGE; 2013.
47. Bech P, Olsen LR, Kjoller M, Rasmussen NK. Measuring well-being rather than the absence of distress symptoms: a comparison of the SF-36 Mental Health subscale and the WHO-Five Well-Being Scale. Int J Methods Psychiatr Res. 2003;12(2):85-91.
48. Ellervik C, Kvetny J, Christensen KS, Vestergaard M, Bech P. Prevalence of depression, quality of life and antidepressant treatment in the Danish General Suburban Population Study. Nordic Journal of Psychiatry. 2014;68(7):507-12.
49. Bech P, Lunde M, Bech-Andersen G, Lindberg L, Martiny K. Psychiatric outcome studies (POS): does treatment help the patients? A Popperian approach to research in clinical psychiatry. Nord J Psychiatry. 2007;61 Suppl 46:4-34.
50. Lane C, McCrabb S, Nathan N, Naylor P-J, Bauman A, Milat A, et al. How effective are physical activity interventions when they are scaled-up: a systematic review. International Journal of Behavioral Nutrition and Physical Activity. 2021;18(1):16.
51. Howlett N, Trivedi D, Troop NA, Chater AM. Are physical activity interventions for healthy inactive adults effective in promoting behavior change and maintenance, and which behavior change techniques are effective? A systematic review and meta-analysis. Transl Behav Med. 2019;9(1):147-57.
