# Supplementary material for: Effectiveness and theory-based evaluation of a personalised digital intervention (EviBody®) for healthy and sustained lifestyle behaviours and well-being among adults: Study protocol for a real-world quasi-experimental study
Source: PLoS One. 2025 Oct 7;20(10):e0333201. doi: 10.1371/journal.pone.0333201 (PMC12503243; doi:10.1371/journal.pone.0333201)
Supplement: S5 File — (PDF) [file pone.0333201.s005.pdf]

Utvärdering av en individbaserad digital tjänst för en hälsosam och hållbar livsstil och välbefinnande

2023-06246-01
Grundansökan
Grundansökan, Medicinteknik/Medicinteknik för in vitro-diagnos k Avslutad

Jenny Louise Rossen

1.2 Ansvarig huvudman för forskningen (forskningshuvudman)

Sophiahemmet Högskola (802006-8741)

1.3 Behörig företrädare för forskningshuvudman

Mari Lundberg

1.3.1 Behörig företrädare – titel som innebär ett verksamhetsansvar

Prefekt

1.4 Har projektet fler forskningshuvudmän?

Ja

1.4.1 Övriga forskningshuvudmän som deltar i projektet:

Karolinska Insitutet (202100-2973)

1.5 Hemvist för forskningen

Institutionen för hälsofrämjande vetenskap

1.6 Huvudansvarig forskare för projektet (kontaktperson)

Jenny Rossen

1.6.1 Institution/hemvist som huvudansvarig forskare är verksam vid

Institutionen för hälsofrämjande vetenskap

1.7 Är den huvudansvariga forskaren disputerad?

Ja

1.8 Andra medverkande:

Unn-Britt Johansson

Philip Von Rosen

3.1 Skriv en populärvetenskaplig sammanfattning av forskningsprojektet.

Bakgrund och syfte
Forskning om digitala tjänster som stöd för hälsosamma levnadsvanor har hittills fokuserat på korta kontrollerade prövningar och det är oklart hur väl de fungerar utanför kontrollerade forskningssammanhang. En utmaning med digitala tjänster är avtagande användarengagemang efter ett par månaders användning. En annan utmaning är att anpassa tjänsten till olika målgrupper.
En digital tjänst (app) som stödjer goda levnadsvanor, är individanpassad med understöd av artificiell intelligens och utformad för ett bibehållet engagemang och hållbara vanor har utvecklats och släppts på marknaden. Tjänsten är framtagen i ett samverkansprojekt med produktutvecklare, forskare och hälsoprofessioner, den är baserad på vetenskaplig evidens och har pilottestats i två genomförbarhetsstudier. Detta projekt syftar till att utvärdera inverkan av den individanpassade tjänsten på välbefinnande hos den vuxna befolkningen. Ytterligare syften är att beskriva spridning och användning av tjänsten i olika grupper i befolkningen, att utvärdera inverkan på måluppfyllelse, beteendeförändring och mental hälsa, samt att utforska faktorer som predicerar eller stödjer beteendeförändring.

Metod
Den digitala tjänsten är en konsumentprodukt och projektet är verklighetsförankrat. Personer som registrerar sig för tjänsten informeras om forskningsstudien och inbjuds att samtycka till att dela sina data till forskningsändamål. Totalt kommer 5000 individer att inkluderas. Ett flertal studiedesigner kommer att tillämpas för att besvara projektets frågeställningar. Data kommer att samlas in med hjälp av enkäter och statistik från appen. Genom en kvasi-experimentell studie med en kontrollgrupp utvärderas det primära utfallet välbefinnande, uppmätt med hjälp av WHO5 Välbefinnadeindex, och utfall på självrapporterad motivation, matvanor, fysisk aktivitet och mental hälsa.

Samhällsrelevans och nyttiggörande
Projektet utvärderar om en digital tjänst har inverkan på välbefinnande, beteendeförändring och mental hälsa utanför den kontrollerade forskningsmiljön. Dessutom avser projektet a öka kunskap och förståelse för hur digitala tjänster ska u ormas för a vara personligt anpassade och för a öka långsiktigt engagemang och beteendeförändring

3.2 Vad är det vetenskapliga sy et med projektet?

Detta projekt syftar till att utvärdera inverkan av en forskningsbaserad individanpassad digital tjänst på välbefinnande hos den vuxna befolkningen. Ytterligare syften är att beskriva spridning och användning av tjänsten, att utvärdera inverkan på måluppfyllelse, beteendeförändring och mental hälsa, att utforska faktorer som predicerar eller stödjer beteendeförändring, samt att beskriva användares perspektiv på tjänsten.

Forskning om digitala tjänster som stöd för hälsosamma levnadsvanor har hittills fokuserat på korta kontrollerade prövningar och det är oklart hur väl de fungerar utanför kontrollerade forskningssammanhang. En utmaning med digitala tjänster är avtagande användarengagemang efter ett par månaders användning och mer kunskap behövs om vad digitala tjänster ska

innehålla för att utgöra ett långvarigt stöd. Ofta upplevs tjänsterna som generella och inte individanpassade. Eftersom alla människor är unika, med unika preferenser, omständigheter, färdigheter och möjligheter behövs mer kunskap om hur digitala stöd kan anpassas och personcentreras utifrån användaren.
Detta projekt inkluderar vuxna personer som använder en tjänst som beteendestöd för fysisk aktivitet, hälsosamma matvanor och mentalt lugn och som samtycker till att dela sin data från tjänsten till forskning. Användare som bryter mot tjänstens användarvillkor kommer att exkluderas. Data från personer som använder ett annat digitalt beteendestöd eller som skattar sitt välbefinnande högt redan vid start exkluderas från analyserna för huvudsyftet.

3.3 Vilka är de vetenskapliga frågeställningarna?

- Förbättrar en digital tjänst med tre nivåer av stöd, utvecklad för att ge individanpassat stöd förhälsosamma vanor, välbefinnandet efter 6 månader jämfört med en kontrollgrupp?
- Hur ser mönstret för användarengagemanget ut under de första sex månadernas användning (vem använder vilka funktioner, när och hur ofta)? Vilka anpassningar görs av den digitala tjänsten och av vilka skäl?
- Bidrar den digitala tjänsten till att uppnå självuppsatta mål, förbättra matvanor, fysisk aktivitet och mental hälsa bland användare efter 6 månader?

- Vilka sociodemografiska faktorer är förknippade med att uppnå de självuppsatta målen och förbättringar av matvanor, fysisk aktivitet och mental hälsa?
- Bidrar de tilltänkta mediatorerna (användarengagemang i tjänsten, ökad motivation, förbättrad self-efficacy och färre upplevda hinder) till effekterna av den digitala tjänsten (måluppfyllelse, förbättrade matvanor och fysisk aktivitet, mental hälsa och välbefinnande)?
- Hur är mönstret för användarengagemang under 24 månader (vem använder vilka funktioner , när och hur ofta)? Vilka anpassningar görs av den digitala tjänsten och av vilka skäl?
- Förbättrar den digitala tjänsten matvanor och fysisk ak vitet, mental hälsa och välbefinnande bland användare under 24 månader?

4.1 Redogör för metod inkl. proceduren, tekniken eller behandlingen.

Bilaga 2, Clinical Investigation plan (CIP), beskriver projektet i detalj inklusive referenser.

Intervention
Tjänsten som ska utvärderas är mobilapplikationen (appen) LongLife Active®. Tjänsten innehåller tre centrala ämnen: 1) God hälsosam mat 2) Fysisk ak vitet och 3) Mentalt lugn. Bilaga 3, Overview functions and BCTs, visar tjänstens interventionskomponenter och beteendeförändringstekniker. Tjänsten innehåller funktioner såsom egenregistrering av vanor, målsättning, handlingsplan, databaser med recept, träningsövningar, avslappningsövningar och kunskapsartiklar, webbseminarier, liveevenemang, justerbara aviseringar och gamification (bl.a. belöningar), socialt stöd genom grupper och individuell- och gruppcoahing.

Procedur
Data kommer att samlas in genom statistik över användning av den digitala tjänsten och med hjälp av frågeformulär direkt i tjänsten. Utöver det samlas dagligt antal steg in från

försökspersonens telefon (via tjänsten) och en aktivitetsmätare skickas till ett slumpvis urval deltagare. Bilaga 4 visar frågeformulären med frågor och svarsalterna. Enkäterna är validerade för populationen, eller är frågor som används inom hälso- och sjukvården. Vissa frågor får alla som använder appen och vissa är studiespecifika. Påminnelser om a svara på enkäterna kommer att skickas veckovis i 4 veckor tills försökspersonen har svarat (max 4 påminnelser). Enkäterna skickas ut vid studiestart och efter 3, 6, 12, 18 och 24 månader, förutom frågor om välbefinnande och tilltro till måluppfyllelse som skickas en gång/månad. Vid studiestart är frågor om demografi inkluderade. Det tar ca 20-30 minuter att svara på frågorna och man kan pausa och fortsätta senare. Aktivitetsmätaren skickas till försökspersonerna tillsammans med instruktioner och en logg för att skriva ner när den tas på och av under dagen. Att svara på frågeformulären och bära aktivitetsmätaren är frivilligt, och om försökspersonen inte svarar kommer det inte a påverka hans/hennes användning av appen.

Data
Demografi: ålder, kön, sysselsättning, yrke, utbildning, ursprungsland och bostadsområde, samt en fråga om hur försökspersonen fick information om tjänsten kommer att samlas in vid studiestart.

Användarmönster kommer att summeras per vecka och beskrivas för kärnkomponenterna i tjänsten. Användarengagemang summeras som antal veckor med engagemang i tjänsten under perioden. Data över anpassning av tjänsten kommer att beskrivas som en sammanställning av betydande uppdateringar och en motivering till varför de görs. Kvalitativa data sammanställs från tjänstens chat-funktion och genom kontinuerlig dialog (mejl, mötesanteckningar) med produktutvecklare. Detaljerad beskrivning finns i bilaga 4. Data över tjänstens användbarhet samlas in med enkäterna System Usability Scale (SUS) och Acceptability by Mobile Application Rating Scale Instrument for Evidence-Based Evaluation (MARS).

För välbefinnande används WHO-5 Välbefinnande Index med total poäng på 100. Välbefinnade är studiens huvudutfall och med en skillnad på 12 poäng mellan interventionsgrupper och kontrollgrupp avses en kliniskt relevant effekt ha uppnåtts. En fluktation i välbefinnande anses vara normalt, försökspersoner med välbefinnandepoäng >70 förväntas inte förbättras och kommer därför att exkluderas från analysen.
Mediatorer för beteendeförändring uppskattas genom The readiness to change questionnaire (RTCQ), the General Self-Efficacy scale (S-GSE) anpassad till beteendeförändring och CDC Road to health barriers. Måluppfyllelse mäts genom statistik över antal dagar/vecka de självsuppsatta målen har registrerats som uppnådda. Tilltro till måluppfyllelse mäta genom en skala 1 låg tilltro till 6 hög tilltro.

Stress mäts med Karolinska Exhaustion Disorder Scale, återhämtning med the Oldenburg Burnout Inventory och sömn med Insomnia Severity Index och självrapporterat antal timmars sömn/dygn. Vidare har fyra studiespecifika frågor tagits fram i samarbete med psykolog för att mäta återkoppling. Försökspersoner som valt sömn som beteendeförändring får också en sömndagbok som vi avser att hämta data från.

Försökspersoner som valt fysisk aktivitet eller matvanor som beteendeförändring får frågor om träning och motion respektive om mat från Socialstyrelsens frågor om levnadsvanor, med tillägg

av frågor om aktiv transport, fysisk aktivitet i yrket och växtbaserade livsmedel. För försökspersoner som valt fysisk aktivitet som beteendeförändring avses vidare att mäta fysisk aktivitet med aktivitetsmätare hos ett slumpvis urval (300 personer).

4.2 Redogör för på vilket sätt metoden skiljer sig från klinisk rutin eller den ordinarie behandlingen.

Den digitala tjänsten finns som en konsumentprodukt, den kan tillhandahållas genom arbetsgivare, men den kan också rekommenderas av hälso- och sjukvårdspersonal, t.ex. genom förskrivning av fysisk aktivitet på recept.
Tjänsten kan komplettera rådgivning vid ohälsosamma levnadsvanor och egenvård vid sjukdomar såsom typ 2 diabetes och högt blodtryck. Idag finns inte något motsvarande stöd generellt inom hälso- och sjukvården. Rådgivande samtal vid ohälsosamma levnadsvanor, egenvårdsutbildning och fysisk ak vitet på recept bygger på samma evidensgrund men är o a inte så omfattande. Det som framförallt skiljer sig är frekvensen av professionellt stöd som tjänsten kan ge, socialt stöd för den som vill, samt a allt är samlat i en app.

4.3 Redogör för tidigare erfarenheter (egna och/eller andras) av den använda proceduren, tekniken eller behandlingen.

Medverkande prövare har under drygt två år tagit fram produkten, samt genomfört två pilot/genomförandestudier. I hela utvecklingsprocessen, som varit en iterativ process, har en mycket nära dialog skett med forskargruppen. Utifrån genomförandestudierna har en separat modul av systemet byggts upp för forskningsdata, samt har en prövarhandbok (bilaga 5) och en "monitoring plan" tagits fram.
Ansvarig i prövargruppen, Julia Bergevi har en master i nutritionsvetenskap. I prövargruppen finns också två hälsopedagoger (med examen från Gymnastik- och idrottshögskolan), en it arkitekt och en produktutvecklare.
Projektet kommer fortsatt att vara ett mycket nära samarbete med forskargruppen. I forskargruppen finns samlat mycket god erfarenhet av interventioner med beteendestöd för levnadsvanor och egenvård.

Jenny Rossen, Med Dr, Sophiahemmet Högskola, principal investigator har erfarenhet bl.a. som projektledare för en komplex RCT om stöd för fysisk aktivitet i primärvården, och god erfarenhet av praktiskt arbete med livsstilsförändringar.
Unn-Britt Johansson, professor, Sophiahemmet Högskola har gedigen erfarenhet av att utforma och utvärdera RCT studier och att använda frågeformulär. Hon har lång forskningserfarenhet av att använda tekniska verktyg för behandling och beslutsstöd.
Maria Hagströmer, professor, Karolinska Institutet har lång forskningserfarenhet av utvärdering av innovativa metoder för a stödja livsstilsförändringar, samt är en erfaren forskare inom mätmetodik, främst inom fysisk ak vitet. Hon har också mycket god erfarenhet av praktiskt arbete med att stödja hälsosamma livsstilsvanor i olika befolkningsgrupper.
Philip von Rosen, docent, Karolinska Institutet har forskningserfarenhet från epidemiologiska och interventionsstudier och gott sta statistikt kunnande.

Susanne Andermo, Med Dr, Karolinska Ins tutet har forskningserfarenhet inom folkhälso- och omsorgsvetenskap, med inriktning på hälsofrämjande insatser och med olika metodologiska ansatser såsom implementeringsforskning.

5.1 Förväntat startdatum för projektet:

2023-11-01

5.2 Förväntat slutdatum för projektet:

2027-10-31

5.3 Tidsplan för de olika delar som ingår i projektet:

Vinter 2023-2024, rekrytering påbörjas
Från och med godkänd etikprövning tillfrågas nya användare av tjänsten a vara med i forskningsprojektet. Rekrytering av kontroller sker samtidigt genom annons i sociala medier. Enkäter skickas ut i tjänsten till användare som ge sitt samtycke till forskningsstudien och via mejl till kontroller. Detta sker löpande vartefter personerna samtyckt.

2024
Fortsatt rekrytering av försökspersoner.
Delstudier som utvärderar inverkan på huvudutfallet välbefinnande, utfall på vanor och mental hälsa, samt processutvärdering för att studera användargrupper och användarmönster under de första 6 månaderna påbörjas. Data samlas in löpande och modeller för statistiska analyser testas.

2025
Fortsatt rekrytering av försökspersoner.
Data för delstudier om determinanter och medierande faktorer hämtas och analyseras.

2026
Data över implementering och effekt efter 24 månader insamlas och analyseras.

Bilaga 6 visar en översikt över planerade delstudier och manuskript.

6.1 Redogör för datainsamling och datas karaktär.

Enkäter skickas ut vid studiestart och e er 3, 6, 12, 18 och 24 månader, förutom frågor om välbefinnande och tilltro till måluppfyllelse som skickas en gång/månad. Vid studiestart är frågor om demografi inkluderade. Data kommer också a samlas in genom sta s k över användning av den digitala tjänsten och med hjälp av frågeformulär direkt i tjänsten. Utöver det samlas dagligt antal steg in från försökspersonens telefon (via tjänsten) och en aktivitetsmätare skickas till ett slumpvis urval deltagare. Bilaga 4 visar frågeformulären med frågor och svarsalternav.

Datats karaktär är blandat, med framförallt självrapporterade skalor som summeras som index eller score.
Dagligt antal steg hämtas från appen som i sin tur hämtar dem från telefonen.

För försökspersoner som valt fysisk ak vitet som beteendeförändring avses a mäta fysisk ak vitet med aktivitetsmätare hos ett slumpvis urval (300 personer). Mätaren är en acceclerometer motsvarande Acti Graph GT3X eller Fibion Sense. Det är en liten platta som fästs på låret och bärs under en vecka. Data som avses samlas in är tid (minuter) i olika aktiviteter (låg-, måttlig-, högintesiv fysisk aktivitet och stillasittande).
Data från sömndagböcker avses samlas in för personer som valt mentalt lugn som område.

Statistik över användning av appen summeras över veckor. Kvalitativa data sammanställs från tjänstens chat-funk on och genom kontinuerlig dialog (mejl och mötesanteckningar) med produktutvecklare.

6.2 Redogör för det statisktiska underlaget för studiepopulationen/ undersökningsmaterialets storlek.

POWERBERÄKNING
Stickstorlek beräknades för a detektera en effekt på 12 poäng baserat på antagandet om tvåsidig testning, α=0.05, power 1−β=0,90 och sd=19.9. För a upptäcka en effektstorlek på 0.5 (Cohens d) för det primära välbefinnandet efter 6 månader rekommenderas en provstorlek på minst 25 försökspersoner per grupp. Förväntat avhopp fram till sex månader är 75 %. Baserat på de förväntade avhoppsfrekvenserna och på att utesluta 20 % från analysen på grund av baslinjepoäng >70, måste vi rekrytera minst 500 försökspersoner för att säkerställa 25 försökspersoner i varje interventionsarm. I kontrollgruppen utgår vi från avhopp och att försökspersoner utesluts ur analysen för att de använder en digital produkt för beteendeförändring och strävar efter att inkludera 200.
De sekundära resultaten är utforskande på grund av tjänstens personcentrerade utformning och begränsad tidigare forskning. Vi siktar på att inkludera 5000 forskningspersoner för att kunna besvara alla forskningsfrågorna.

MOTIVERING TILL POWERBERÄKNING FÖR PRIMÄRT UTFALL
Det primära utfallet utvärderas med WHO-5 Välbefinnande Index som anges i en skala 0-100 (Topp et al 2015). I vår genomförandestudie var medelpoängen vid baslinjen 55. Instrumentet har såvitt vi vet inte tillämpats på en generell svensk befolkning tidigare. I danska befolkningsundersökningar var den genomsnittliga WHO-5-poängen 60-70 (sd=19,0-19,9) (Bech et al 2003, Ellervik et al 2014, Bech et al 2018). En förändring på 10-20 poäng i WHO-5 har föreslagits som en kliniskt relevant förändring (Bech et al 2007, Bech et al, 2018). I uppskalade insatser förväntas 60 % lägre effekter (Lane et al 2021). Vi siktar därför på en skillnad på 12 poäng i poängen mellan interventionsgrupper och kontrollgrupp samt mellan baslinje och 6 månader. En fluktuation i välbefinnande anses vara normal (Bech et al 2018), försökspersoner med välbefinnandepoäng >70 förväntas inte förbättras och kommer att exkluderas från analysen. I pilotstudien nådde 21 % av deltagarna 70 poäng vid baslinjen.

6.3 Hur kommer undersökningsprocedurerna a dokumenteras?

Svar på enkäterna och statistik över användning av tjänstens funktioner sparas direkt i systemet.

6.4 Hur kommer insamlad data att hanteras och förvaras?

Forskningsdata sparas pseudonymiserat i systemet som backar upp tjänsten.

All data lagras i krypterad form och är kategoriserad i tre nivåer med den direkt personligt identifierbara informationen låst i den högsta kategorin. Kategorierna är separerade från varandra och har strikt begränsad åtkomst. All kommunikation i systemet sker över krypterade förbindelser och all information lagras i krypterad form. För personlig information i den högsta kategorin tillämpas dubbel kryptering med individuella nycklar. Delning av känslig data loggas löpande för a följa upp när den hämtas och av vem. Loggarna övervakas även med larm på avvikelser.

Under pilotstudierna togs syntax och algoritmer fram, testades och förfinades för a skapa Excelfiler med datat. Kodning görs genom a försökspersonerna får e ID när de samtycker till forskningsstudien. ID genereras av systemet via lokala löpnummer som är kopplade till den aktuella studien. Systemet tillhandhåller en separat modul för studier som har till uppgift att hantera studier och studiedeltagare. Det möjliggör att användare kan delta i studier via systemet och a systemet kan delge data på individnivå utan a dela någon personligt identifierbar information.

Ansvariga forskare hämtar data i form av Excel-filer från en hemsida genom a legitimera sig digitalt. Datat sparas sedan på Sunet Drive för Sophiahemmet Högskola under 15 år. Kodlista med namn och mejladress hämtas och förvaras avskilt från de andra uppgifterna och sparas på en lösenordskyddad plats.

7.1 Vilka risker kan ett deltagande medföra för de forskningspersoner som ingår i forskningsprojektet?

Forskningspersonerna anmäler sig till den digitala tjänsten i syfte att använda den som en konsumentprodukt, inte för att vara med i en forskningsstudie. Vid registrering i appen eller visat intresse från annons visas en förfrågan om att delta i forskningsstudien med information om studien (bilaga 7a och b Forskningspersonsinforma on). Personer som samtycker till att dela sina data för forskningsändamål tillfrågas att besvara frågor som har tillhandahållits av forskare. Detta kan orsaka stress och eventuellt distraktion från användarens avsedda användning av tjänsten, samt ökad oro för privat integritet då uppgifterna kommer att överföras till forskarna. A samtycka till a delta i en forskningsstudie kan också leda till en känsla av commitment, att man måste prestera. Vid beteendeinterventioner finns risk för dåligt samvete och minskat självförtroende om man inte når sina uppsa a mål och för överdrivet beteende som i extrema fall kan leda till ätstörningar och orthorexi.

A besvara frågor om si mående och sina vanor kan innebära a man reflekterar över sin hälsa och livssituation vilket kan leda till oro och stress. Livsstillsförändringar kan också innebära förbättringar i hälsan som leder till att man kan behöva sänka dosen av eventuella mediciner. Det är därför viktigt att ha en dialog med behandlande läkare och kännedom om att om man återgår till den tidigare livsstilen kan man behöva öka dosen igen.

I tjänsten ingår socialt stöd genom chatgrupper. I dessa grupper finns risk för en känsla av grupptryck, nedlåtande kommentarer, missförstånd och a icke saklig information sprids.

7.2 Vilken nytta kan ett deltagande medföra för de forskningspersoner som ingår i forskningsprojektet?

För både interventionsgrupperna och kontrollgrupperna kan enkäterna med frågor innebära en reflektion över den egna livsstilen och ökad motivation att ta kontroll över sin egen hälsa.

Den förväntade kliniska fördelen med regelbunden användning av den digitala tjänsten är förbättrat välbefinnande, förbättrade levnadsvanor och mental hälsa (minskad upplevd stress). Ytterligare fördelar som e individanpassat stöd för att förbättra sin livsstil kan leda till, även om de inte mäts i studien, minskade depressiva symtom och förbättrade kardiometabola riskfaktorer (t.ex. övervikt, blodfetter, metabol kontroll och blodtryck).

7.3 Gör en värdering av förhållandet mellan riskerna och nyttan av projektet.

De förväntade fördelarna med den digitala tjänsten uppväger de potentiella riskerna. I första hand finns de förväntade fördelarna med ökat välbefinnande och livsstilsförändring. Utöver dessa har tidigare studier med digiala interventioner rapporterat om en ökad känsla av egenmakt och ökad hälsoliteracitet. Exempel på fördelar är förbättrade beslutsfärdigheter som rör den egna hälsan, upplevt förbättrad egenvård, förståelse för egenmätning, ökad medvetenhet, en känsla av kontroll över si hälsotillstånd och förbättrad motivation att fortsätta med egenvård. Alla dessa effekter överväger riskerna med deltagande.

Själva tjänsten som utvärderas är framtagen för a minska den upplevda hälsan och stödja varje individ utifrån dennes situation. I studien kommer försökspersonerna att sätta upp egna mål och själva bestämma en veckoplan för hur de ska nå målen i sin egen takt. Förslag, uppmuntran, inspiration och feedback individanpassas för varje individs preferenser och identifierade behov. Dessa inbygggda åtgärder minskar riskerna med deltagande och en eventuell känsla av commi ment för a det är en forskningsstudie.

7.4 Beskriv hur projektet har utformats för att minimera riskerna för forskningspersonerna.

Risker övervakas, monitoreras och hanteras systema sk genom en "monitoring plan" och processerna följer ISO 14155. Riskerna för dataosäkerhet hanteras med stor försik ghet. Systemet är byggt för a aldrig dela några personliga uppgi er med någon part, och llämpar pseudonymisering med anonyma ID-nummer för alla användare. Personuppgi er kommer a lagras i e dubbelkrypterat kategoriseringssystem i tre nivåer för a säkerställa datasäkerhet och personlig integritet genom a separera personligt iden fierbar informa on från personligt känslig informa on. Systemet har strikt begränsad llgång ll personuppgi er med reglerade, övervakade och loggade funk oner som möjliggör åtkomst ll begränsad och anonym data endast för behöriga. Till exempel måste auktoriserade forskare och hälsocoacher iden fiera sig med elektronisk iden fiering (BankID) för a få llgång ll strikt begränsade och anonyma uppgi er, endast relevanta för forskning eller hälsocoaching. Systemet uppfyller gällande krav på säkerhet och övervakning, vilket säkerställs genom regelbundna säkerhetskontroller.

Den digitala tjänsten ska kunna känna av och varna om personer har e beteende som kan vara alarmerande u från de de kända riskerna. Coacherna som kommer a användas genom tjänsten ska alla ha minst 7.5 hp inom beteendeförändring på högskolenivå, samt genomgå en utbildning som är
specifik för tjänsten. Deras roll är a stö a u från individens behov, hälsostatus, preferens och digare erfarenheter. Coacherna och även systemet tränas i a iden fiera signaler om deltagare inte mår bra och kan fånga upp dem och hänvisa ll lämplig vård. Coaherna modererar gruppcoaching och övervakar gruppcha ar.

Deltagarna kommer a påminnas om a deltagandet forskningsstudien är frivilligt, a de närsomhelst kan avbryta si deltagande utan a ange orsak och a de inte ska känna någon press a vare sig förändra si beteende u från a det är e forskningsprojekt.

7.5 Identifiera och precisera om eventuella etiska problem (nackdelar/fördelar) kan uppstå i ett vidare perspektiv genom forskningsprojektet.

Det finns en överhängande risk a urvalet med personer som samtycker till deltagande i forskningstudien inte speglar den svenska vuxna befolkningsstrukturen och a kunskapen därmed inte blir tillämplig på alla grupper i samhället. Den digitala tjänsten, och liknande produkter, innebär en kostnad för användaren, den kräver tillgång till en smartphone och internet, förmåga a förstå det svenska språket ganska bra och viss digital literacitet. Utöver det är den utvecklad baserad på forskning u från "main stream" populationer, i huvudsak från höginkomstländer, o a med hög andel högutbildade, kristna, hälsomedvetna och engagerade individer.

Det finns en stor risk att både tjänsten och kunskapen som genereras inte kommer till nytta för vissa grupper i befolkningen. Detta kan bidra ytterligare till hälsoklyftorna i samhället och ojämlik vård.

8.1 Hur görs urvalet av forskningspersoner?

Alla vuxna som registrerar sig i tjänsten LongLife Active® inbjuds at vara med i forskningsstudien. Eftersom den är verklighetsbaserad är vi intresserade av alla personer som nås av tjänsten. Till kontrollgruppen inkluderas alla vuxna som samtycker till att besvara enkäter. Om vi identifierar släkt, vänner eller kollegor till forskare eller ägare av appen tas data från dessa personer inte med i analyserna eftersom de kan tillföra bias.

8.2 Hur många forskningspersoner kommer att inkluderas i forskningsprojektet?

Vi har som mål a samla data från totalt 5000 personer. För de första delstudierna som studerar tjänstens inverkan inkluderas de första 500 som samtyckt till forskningsstudien. Till kontrollgruppen inkluderas 200 personer.

8.3 Vilka urvalskriterier kommer att användas för inklusion?

Interventionsgrupper
Vuxna ≥18 år som registrerar sig för tjänsten LongLife Active®, identifierar sig med BankID och samtycker till forskningsstudien.

Kontrollgrupp
Vuxna ≥18 år som ser annons om forskningsstudien och samtycker till att svara på enkäter.

8.4 Vilka urvalskriterier kommer a användas för exklusion?

Interventionsgrupper
Personer som bryter mot tjänstens användarvillkor
Data från personer med >70 poäng på WHO-5 Välbefinnande Index exkluderas från analyser av det primära utfallet välbefinnande. Personer som har bekant-eller släktskap med forskare eller ägare till tjänsten utesluts ur analyserna.

8.5 Ange relationen mellan forskare och forskningspersonerna.

Det finns inga relationer mellan forskare och försökspersonerna.

8.6 Vilket försäkringsskydd finns för de forskningspersoner som deltar i forskningsprojektet?

Försökspersonerna använder tjänsten som en konsumentprodukt och företaget LongLife Ac ve ansvarar för försäkring för sina kunder. Forskningsstudien täcks av Sophiahemmets olycksfallsförsäkring.

8.7 Redogör för den beredskap som finns för att hantera oväntade bifynd eller händelser under forskningsprocessen som kan äventyra forskningspersonernas säkerhet.

Hög upplevd stress, överdrivet beteende, ångest, en känsla av misslyckande, hopplöshet och låg självkänsla mildras genom stöd genom att sätta upp realistiska och progressiva mål, uppmuntran, information och webbseminarier om beteendeförändringar och hälsosamma vanor. Varnings- och rapporfunktioner är inbyggda i systemet för att larma överdrivna vanor och lågt välbefinnande. Vid välbefinnandeindex poäng 50 eller lägre får försökspersonen ett meddelande om att det är lågt, att det är normalt att skatta sitt välbefinnande lågt under en kort tid, men också hänvisning till lämplig vård om man upplever sig fortsatt ha lågt välbefinnande. Med i projektet finns ett team med hälsocoacher och psykologer som följer upp överdrivna vanor, vid t.ex. dagligt antal steg >26 000 kommer ett meddelande om att man har en väldigt hög nivå av fysisk aktivitet, a man också behöver lägga in vilodagar och rekommendation om a boka ett hälsocoachsamtal om man önskar för att diskutera hur man kan få en balans i sin aktivitet. Alla meddelanden sker i en trevlig och tillmötesgående ton som ska signalera att det är ett stöd, inte en kontrollfunktion.

8.8 Kommer ekonomisk ersättning eller andra förmåner betalas ut till forskningspersonerna?

Nej

9.1 Kommer forskningspersonerna a informeras om forskningsprojektet och tillfrågas om de vill vara med eller inte?

Ja

9.1.1 [Om Ja 9.1] Hur, när (i vilket skede) och av vem informeras och llfrågas forskningspersonerna?

Personer som registrerar sig för den digitala tjänsten LongLife Ac ve informeras om forskningsstudien direkt i tjänsten och tillfrågas om de vill delta. Samtycke till deltagande kan ENDAST göras e er a man har läst informationen (bilaga 7a) och genom a identifiera sig med hjälp av BankID. Det framgår tydligt att man när som helst har möjlighet att avbryta sitt deltagande utan att uppge varför och hur man enkelt kan göra det vid ett senare skede. I tjänsten kommer man då och då a bli påmind om att det är en pågående forskningsstudie och delges forskningsrön.

Kontroller rekryteras via sociala medier och genom en förfrågan via stora arbetsgivare för att få en spridning till lågsocioekonomiska grupper. Till personer som är intresserade av att vara med skickas den detaljerade forskningspersonsinformationen (bilaga 7b) via e-post. Kontrollpersoner samtycker i enkätverktyget innan de börjar svara på första enkäten. Innan de samtycker kan de återigen läsa igenom informationen.

9.2 Kommer barn under 18 år a ingå i forskningsprojektet?

Nej

9.3 Kommer forskningspersoner, vars mening på grund av sjukdom, psykisk störning, försvagat hälsotillstånd eller något annat liknande förhållande inte kan inhämtas, att ingå i forskningsprojektet?

Nej

10.1 Kommer projektet att begära ut uppgifter från ett befintligt register?

Nej

11.1 Finns det relevanta resultat från djurförsök?

Ej aktuellt

12.1 Hur garanteras tillgång till data för forskningshuvudmannen och medverkande forskare?

Data sparas löpande på Sunet Drive för Sophiahemmet Högskola. Data är endast tillgängligt för forskare inom projektgruppen vid Sophiahemmet Högskola. Vid behov delas data till de medsökande forskarna vid Karolinska Ins tutet. I sådana fall upprättas avtal för dataöverföring. Samarbetsavtal mellan lärosäten finns. Dataöverföringsavtal skrivs vartefter utifrån aktuella studier och behov.

12.2 Vem eller vilka ansvarar för databearbetning och skriftlig redovisning av resultaten?

Doktorander och forskare anställda vid Sophiahemmet Högskola och Karolinska Ins tutet kommer att bearbeta och analysera data under överseende av PI Jenny Rossen vid Sophiahemmet högskola.

12.3 Hur och när planeras resultaten a offentliggöras?

Resultaten planeras att presenteras vid nationella och internationella konferenser, samt genom publicering i vetenskapliga tidsskrifter med open access som tillämpar peer review. Bilaga 6 visar planerade delstudier och tilltänkta inkluderade forskare. Publicerade resultat förmedlas genom korta populärvetenskapliga texter och videos till deltagarna i appen.

12.4 På vilket sätt garanteras forskningspersonernas rätt till integritet när materialet offentliggörs?

Dataredovisning sker endast på gruppnivå.

13.1 Redovisa eventuella ekonomiska överenskommelser med bidragsgivare eller andra finansiärer (namn och belopp).

Ingen överenskommelse har slutits med företaget Longlife Ac ve AB som distribuerar den digitala tjänsten gällande finansiering av arbetskostnader i forskningsprojektet för medverkande forskare. Forskargruppen söker externa forskningsbidrag.

13.2 Redovisa forskningshuvudmannens, huvudansvarig forskares och medverkande forskares egna ekonomiska intressen.

LongLife Active AB står för produktutvecklingen och ingen ekonomisk ersättning har eller kommer att utgå från forskningsprojektet till företaget.
Ingen av de enskilda forskarna har ägarskap i företaget LongLife Ac ve eller på annat sä ekonomiskt vinstintresse av projektet, har ej och kommer inte a avlönas av företaget. Vid gemensamma ansökningar om medel, t.ex. till Vinnova, kommer parterna att uppges enskilt och det kommer tydligt a specificeras vilka kostnader som söks för respektive part.

Forskningsplan

Den sammanfattande beskrivningen av forskningsprojektet ska förstås av fackmän. Den kan lämpligen utformas enligt följande:
Vetenskaplig frågeställning: En redogörelse för det övergripande sy et med det föreslagna forskningsprojektet samt specifika mål (primära och sekundära frågeställningar).
Områdesöversikt: Ge e sammandrag av egna och andras forskning och tidigare resultat inom forskningsområdet. Översikten ska tydliggöra det aktuella projektets relevans. Nyckelreferenser ska anges.

Projektbeskrivning: Gör en sammanfattning av projektets/motsvarande uppläggning. Urval av forskningspersoner, procedurer, metoder med mera ska tydligt redovisas. Det ska framgå hur metoder, urval och procedurer kan ge svar på de specifika frågeställningarna. Om flera delprojekt avses anges sekvens för genomförande och på vilket sätt de efterföljande delprojekts uppläggning kan bero av resultaten av e föregående.
Betydelse: Ge en kor a ad redogörelse för projektets betydelse för forskningsområdet.
Preliminära resultat: Kan i förekommande fall anges.
SKA VARA PÅ SVENSKA ELLER ENGELSKA.

1._Research_plan.pdf
553.25KB

registrator@etikprovning.se | 010-475 08 00 | Box 2110, 750 02 Uppsala | etikprovning.se

Research plan

Evaluation of an individually tailored digital service aimed to promote healthy and sustainable behaviors and well-being

Abstract

Research problem and specific questions

Research on digital solutions for behavior change has focused on short-term controlled efficacy trials. A major challenge of digital products is high rates of abandonment. A digital service supporting behavior change based on scientific evidence, aided by artificial intelligence, and designed for enhanced engagement has been developed. This project aims to evaluate the impact of the individually tailored digital service on wellbeing among the adult population. Secondary objectives are to describe the internalization process, evaluate the impact of the digital service on reaching selfidentified goals, behavior change, and mental health, explore predicting and mediating factors for a response, and explore users' perspectives of the service.

Data and method

The digital service is a consumer product, and the project is a real-world project. Individuals signing up for the service will be informed about the research study and invited to consent for data extraction until reaching 5000 consenting individuals. Several research designs with a combination of qualitative and quantitative methodologies will be applied. Data will be collected by self-report instruments, analytics from the app, and individual interviews. A quasi-experimental study with a control group will evaluate the primary outcome, well-being, assessed by the WHO-5 Well-Being Index, and change in self-reported motivation, diet, physical activity, and mental health.

Societal relevance and utilization

The project evaluates the effectiveness of an up-scaled digital intervention for behavior change outside the controlled research setting. In addition, the project intends to enhance the knowledge and understanding of how digital services should be designed to be personally tailored and to enhance long-term engagement and behavior change. It will increase the knowledge on how to reach individuals who are usually not reached by health promotive initiatives.

Background

Reducing premature mortality from non-communicable diseases through prevention and treatment, and promoting mental health and well-being are targets in the sustainable development goal 3 of the Agenda 2030. Healthy lifestyle habits are central self-management aspects in preventing and treating widespread noncommunicable diseases, such as diabetes, cardiovascular disease, cancer, and depression (1). Unhealthy lifestyle habits are clearly associated with low well-being (2), and low socioeconomic groups are more susceptible to both unhealthy habits and reduced health than high socioeconomic groups (3).

Digital technologies based on artificial intelligence can provide personalized support for healthy behaviors and empower individuals to actively participate and track their health progress (4). The evidence for positive short-term (<6 months) effects of digital technologies on lifestyle change and health outcomes in controlled settings is rigorous (5, 6), and using self-monitoring and goal setting as fundamental tools in interventions for behavior change is powerfully advised (6, 7). To the best of our knowledge, the impact of digital technologies promoting healthy habits on *well-being* is not wellstudied.

Though, research has until now focused on short-term controlled efficacy trials, and upscaled long-term (>12 months) implementation studies are rare. Real-world trials are needed to establish if digital interventions are effective for behavior change outside the controlled research setting (8). A major challenge of digital products is high rates of abandonment (9, 10) and factors influencing engagement with digital technology need to be understood and overcome (9, 11). Internalization and implementation factors such as reach and dose, as well as determinants and mediators of efficiency, are rarely researched (11, 12). More studies are needed to extend the knowledge on which content and functions of digital services are effective for whom. In addition, perspectives of various subgroups should be explored to adapt the technologies to groups who are less often participating in research projects.

Aims and objectives

The overall aim of the project

The project aims to evaluate the impact of a digital service for healthy habits on wellbeing and explore what functions and contents of the digital service support sustainable behavior change and well-being and are appreciative for various subgroups.

Primary aim

I) The primary objective of this project is to evaluate the effectiveness after 6 months of an individually tailored digital service aimed to support healthy habits among the adult population on well-being compared to a control sample of the general population.

Secondary objectives

II) To study internalization and implementation factors such as reach and dose of the medical device across 6 months.

III) To explore the impact of three different levels of digital support for healthy habits on reaching self-identified goals, dietary and physical activity habits, and mental health after 6 months.

IV) To explore determining response of sociodemographic factors in meeting the selfidentified goal, dietary and physical activity habits, mental health, and well-being.

V) To evaluate the intended mediators (engagement with the service, improved motivation, self-efficacy, and reduced perceived barriers) of the medical device on achieving the self-identified goal, improved dietary and physical activity habits, mental health, and well-being.

VI) To explore what app functions and features mediate improvements of the medical device on behavior change, mental health, and well-being.

VII) To explore the engagement of using the digital service and adherence to behavior change across 24 months.

VIII) To explore the patterns of achieving the self-identified goal, change in motivation, dietary and physical activity habits, mental health, and well-being across 24 months.

Theoretical framework

The project is guided by the Medical Research Council framework for developing and evaluating complex interventions and intends to evaluate effectiveness, for whom the intervention work, and to theorize why the intervention work (13). Figure 1 shows an overview over the the larger project, in which this project is phase 3. In 2020-21 a digital service was developed using a co-development approach including various stakeholders (14). In total 9 workshops were conducted with researchers, product developers, designers, and healthcare professionals along with numerous short meetings in smaller groups in between the workshops. The COM-B system proposing capability, opportunity, and motivation as conditions for behavior served as a structural framework for the workshop discussions (15). The process of tailoring functions and features in the app was guided mainly by the Transtheoretical Model of Change (16). After the development and prototype testing the Behavior Change Technique Taxonomy v1 (17) was applied to label the functions that were finally included in the digital service. Thus, the use of behavior change techniques allows the included functions to be evaluated as mediators, compared to other studies, and included in meta-analyses.

Figure 1. The overall project of developing, refining, and evaluating a digital service for behavior change. This research plan describes phase 3.

Prior research

The project is planned based on an extensive body of earlier research, both external research, and research conducted by the research group (18-21). It builds on the knowledge and experiences obtained from the Sophia Step Study, a three-armed randomized controlled trial evaluating self-monitoring of physical activity and additional counselling as support for self-management of physical activity (19, 22). The results showed a large individual variation, both in baseline behavioral patterns and in the level of behavior change. An interview study revealed that the participants were appreciative of the personalized approach of the program, receiving feedback on health outcomes and positive reinforcement (21). Participating in the research study led to a reported increase in awareness and motivation for physical activity, the establishment of new routines, and a feeling of control over the own health (21). The findings from Sophia Step Study point to the importance of applying an individual approach, targeting individual circumstances, needs, and preferences.

An intervention planning phase was conducted prior to this project based on a framework for developing behavioral interventions (14) and a guide to developing and planning digital interventions (23). This planning phase included a systematic review of users' perceptions of e- and mHealth services promoting physical activity and healthy diets (18) and two qualitative studies describing users' (24) and healthcare professionals' perspectives on digital support for physical activity (submitted manuscript). The findings from the systematic review and the qualitative studies served as the basis for the development of the digital service (the app LongLife Active®).

A pilot study evaluating the feasibility of the digital service LongLife Active® was completed in August 2022. The pilot study aimed to optimize the service to become usable and acceptable based on users' opinions. The subjects tested the service for three months. Validated questions on usability and acceptability of various functions were integrated into the service. The findings from the pilot study are not yet published but have powerfully guided further development and optimization of the digital service as well as the process for data collection.

Method

Study designs

The primary study design of the project is a quasi-experimental study with a control group. A four-armed control design will allow for comparison between a control group, and three levels of intervention. A process evaluation is planned to describe uptake and internalization. The project will also collect data as an observational study to explore patterns, predictors, and mediators in longitudinally. In addition, qualitative interview studies are planned to gather perspectives and views from sub-groups of users.

Recruitment

The digital service will be launched and promoted by the company LongLife Active AB as a real-world project. The ambition is to reach individuals in need of lifestyle change, with a special emphasis on hard-to-reach groups. Employers, benefit platforms (e.g.

Benify and Epassi), health care services, pharmacies, and social media will be targeted channels. Employers may apply for the health promoting subsidy, Friskvårdsbidrag, when employees sign up for LongLife Active®. Health care patients prescribed physical activity (Fysisk aktivitet på recept FAR) from their care provider is allowed a reduced fee on the membership of LongLife Active®.

Individuals who sign up for the service from November 2023, or as soon as ethical approval is in place, will be informed about the research study and invited to consent for data extraction. Controls will be recruited through social media.

Inclusion criteria (intervention arms): Adults (≥18 years) who sign up for the service LongLife Active® using BankID for identification, and consent to the research study.

Exclusion criteria (intervention arms): Age ≤17 years. Individuals who are discharged from the app due to refracting the terms of the service will be excluded. Users scoring >70 on well-being will be excluded from the primary analyses and users who appear as friends, colleagues, or family with anyone in the research or owner group will be excluded from the analyses.

Inclusion criteria (controls): Adults (≥18 years).

Exclusion criteria (controls): Age ≤17 years. Is currently using a digital product supportive for behavior change (e.g. Livsstilsverktyget, Health Integrator, Weight Watchers). Is found to be a user of LongLife Active. Subjects scoring >70 on well-being will be excluded from the primary analyses and subjects who appear as friends, colleagues, or family with anyone in the research or owner group will be excluded from the analyses.
Sample size: The estimation is to reach 5000 consenting individuals using the service and 200 controls.

Recruitment to interviews will be made by purposive sampling. Users who full-fill inclusion criteria (educational level below secondary school and in a low-socioeconomic living area, and profession as blue-collar worker) will be invited to give their viewpoints.

Intervention

LongLife Active® is a mobile phone application aimed to support healthy habits and improve well-being. The service targets three subjects: 1) a Healthy diet, 2) Physical activity, and 3) Mental balance. Seven core intervention components are included;

a) *Onboarding*: tutorials about the service and prompts for commitment to behavior change;
b) *Individual action plan*: self-monitoring of behaviors and outcomes, goal setting, choice of daily or weekly activities, instant feedback on behavior, and feedback on the outcome;
c) *Interaction and feedback*: reminders, notifications, digital rewards, and challenges;
d) *Knowledge and inspiration*: food recipes tailored to goals, exercises tailored to capacity and goals, educational articles, videos, live and recorded skills training webinars (e.g. cocking classes and exercise sessions).
e) *AI-chat*: basic counselling regarding the action plan.
f) *Community:* professional group counseling and peer support.
g) *Individual coaching:* professional counselling.

The levels of intervention are Basic: core functions including onboarding, action planning, interaction and feedback, knowledge and inspiration, AI-chat, Standard: the same core functions + group coaching and community, Premium: the core functions + group coaching, community + individual counseling.

Data collection

Data will be collected by self-report instruments (questionnaires within the app), analytics from app use and individual interviews. *Supplement 4a. Variabellista* shows the included questionnaires and time points for data collection. Kind reminders to answer the questionnaires will be pushed out weekly for 4 weeks until the subject has answered (max 4 reminders).

Reach

Information on promotion channels will be collected from the provider. This data is not connected to the subjects. Demographics: age, gender, occupational status, profession, education, country of origin and living area, as well as a question on how the subject got information about the service will be collected by questions when registering to the digital service.

Fidelity and dose

User activity patterns across time will be aggregated per week and described for the core functions: A*ction plan, Knowledge and inspiration*, *Coaching* and *Community.* Adherence with app use will be estimated using the frequency of weekly use across the tracking period.

Adaptations

Qualitative data from the support service chat, and from continuous dialogues with health coaches, recipe providers and staff maintaining the digital service and records over notable adaptations and changes made to the service will continuously be collected.

Usability

Perceived usability of the service will be collected by the System Usability Scale (SUS) (25) and acceptability by Mobile Application Rating Scale Instrument for Evidence-Based Evaluation (MARS) (26).

Mediating factors

Motivation will be assessed by the readiness to change questionnaire (RTCQ) based on the transtheoretical model (27, 28). Self-efficacy will be evaluated by the General SelfEfficacy scale (S-GSE) adapted for behaviour change (29). Barriers to healthy behavior will be evaluated by the Barriers to being active quiz (CDC Road to health barriers) and Perceived barriers in trying to eat healthier (30).

Primary outcome measure

The primary outcome well-being will be assessed by the WHO-5 Well-Being Index (31). The WHO-5 comprises the following five items: being cheerful and in good spirits; being calm and relaxed; feeling active and vigorous; feeling fresh and rested when waking up in the morning; and having an interest in day-to-day activities. Six response alternatives

are scored from 5 (All of the time) to 0 (At no time). The total raw score which ranges from 0 to 25 is multiplied by 4 to calculate the final score. The final score range goes from 0 = worst imaginable well-being to 100 = the best imaginable well-being.

Secondary outcome measures

Mental health

Stress will be assessed by the Karolinska Exhaustion Disorder Scale, a self-rating scale for stress-induced exhaustion disorder (32). Burnout will be assessed by the Oldenburg Burnout Inventory (OLBI) (33). Sleep will be assessed by the self-reported number of hours of sleep per night and ISI – Insomnia Severity Index (34). Four questions have been developed specifically for this study to assess mental balance.

Goal achievement

Reaching the self-identified goal will be evaluated by collecting information on number of days/week the self-identified goal was met during the past month. Confidence for reaching the goal will also be asked for on a scale 1 low confidence to 6 high confidence.

Dietary habits

Dietary behaviours will be assessed by questions from the Social Board of Health and Welfare (35), with the addition of some study specific questions adapted in accordance with the EAT Lancet commission on healthy diets from sustainable food systems (36).

Physical activity and sedentary behaviors

Physical activity and sedentary behaviors will be collected by questions from the Social Board of Health and Welfare (35) with additional questions on active transport and physical activity at work. In a randomized sample (n=200) physical activity and sedentary time will also be measured objectively by the use of the three-axial accelerometer-based device Fibion Sens (Fibion Inc, Jyväskylä, Finland) (37).

Statistical design and analysis

Figure 2 shows a logic model for the project with the anticipated demographic predictors, mediators (engagement, motivation, self-efficay and reduced barriers), secondary (intermediate) outcomes (achieving self-identified goals and improved behaviors and mental health), and the primary endpoint well-being.

Figure 2. Logic model for the digital service LongLife Active showing the predicted underlying pathway and responses.

Descriptive statistics

Descriptive statistics will be applied to report the reach of the intervention including demographics and user engagement patterns (analytics over time) for intervention dose. In addition, user engagement patterns will be specified for subgroups (age, gender, employment status, profession, educational level, residence in urban, rural, or semirural area and country of origin).

Change in mediators, intermediate outcomes and the endpoint well-being will be reported between baseline and each follow-up time point, presented by intervention group.

Analytical procedures

Change in the endpoint well-being (as total score) and the intermediate outcomes dietary habits, physical activity, and mental health, will be analysed across time and between groups using mixed model for repeated measures, and reported as estimates with confidence intervals. The model will include intervention level and time (0, 6, 12, 18 and 24 months).

To investigate associations of demographic factors and user engagement with behavior change, mental health, and well-being, structural equational modelling and mixed models will be applied.

Mixed models will be applied to evaluate mediating factors for response. These analyses will explore if high levels of motivation, self-efficacy and reduced perceived barriers mediates reaching the self-identified goal, improvements in behavior, mental health, and well-being and whether improvements in behavior and mental health mediate wellbeing. Cut off values for the intermediate outcomes will be derived based on baseline values and findings from similar studies.

The threshold for statistical significance will be set at $p<0.05$.

Qualitative analyses

Qualitative data (chat groups and dialogues with included actors) will be analyzed inductively using manifest content analysis (38).

Sample size calculation

A desirable sample size has been calculated to detect an effect of 12 points on wellbeing (39, 40) based on the assumption of two-tailed testing, an alpha error $\alpha=0.05$, power $1-\beta=0.90$ and sd=19.9 (41). To detect a medium standardised effect size of 0.5 (Cohen's d) for the primary outcome well-being at 6 months a sample size of at least 25 subjects per group is recommended. Expected drop-out rate due to withdrawal and lost to follow-up at six months is 75 %. Based on the expected drop-out rates and on excluding 20% from the analysis due to baseline score >70, we need to recruit at least 500 subjects to assure 25 subjects in each intervention arm. In the control group we assume subjects to be excluded based on using a digital product for behavior change and strive to include 200 subjects.

The secondary outcomes (achieving self-identified goals and improved behaviors) are exploratory due to the person-centred design of the service. The subjects will set individual goals and possibly change goals during the intervention. Likewise, habits and behavior change are largely changeable due to circumstances. The current research is not immense enough to guide a decision for minimal expected effects when individual goals are set (e.g., reducing weekly servings of red meat). A larger sample size than what is required for the primary endpoint well-being is desired. Similarly, the secondary analyses of predicting and mediating factors-require a large sample size. We therefore aim to include 5000 subjects.

Relevance

The ultimate goal of the project is prevention of noncommunicable diseases and improved well-being in the population. Noncommunicable diseases are causing 80% of disabling life and shortening life expectancy, and on the society causing costs for health care and work absenteeism (1). Unhealthy diets and physical inactivity increase the risk of non-communicable disease (1).

Healthcare professionals should advise individuals with unhealthy habits to make better choices. However, behavior change is challenging, and person-centered support and follow-up based on individual preferences, capabilities, and opportunities are required. Healthcare professionals describe barriers to supporting behavior change in terms of lack of time, resources, education, and management backing. Similarly, many employers have the ambition to ease employees to make healthy choices but require tools for health-promotive work.

The recent Covid-19 pandemic accelerated a shift in healthcare delivery, highlighting the need for effective methods for self-management and transition from healthcare to more home-based and digital care. We now have an open window of opportunities for primary prevention and digital health promotion initiatives. Digital services are possible to rapidly scale up nationwide and globally. Besides, by using digital technologies properly and carefully inequalities in health due to low health literacy, social group, type of job, geographic location, gender, age, physical and psychological capabilities, and other discriminating determinants can be diminished and even eliminated.

Empowerment and health literacy benefits of digital interventions have been reported. Examples are improved decisions making skills concerning the own health, perceived facilitation of self-management, an understanding of self-monitoring readings, increased awareness, a sense of control over a condition, and improved motivation to continue self-management activities (42).

This project will shed light on factors enhancing or limiting the impact of digital services over time and in subgroups of the population. Thus, the project will add knowledge to how digital interventions can be optimised and adapted to individuals for whom standard lifestyle advice may be difficult to follow.

References

1. WHO. [Available from: https://www.who.int/data/gho/data/themes/noncommunicable-diseases.
2. Stenlund S, Koivumaa-Honkanen H, Sillanmäki L, Lagström H, Rautava P, Suominen S. Changed health behavior improves subjective well-being and vice versa in a follow-up of 9 years. Health Qual Life Outcomes. 2022;20(1):66.
3. Kraft P, Kraft B. Explaining socioeconomic disparities in health behaviours: A review of biopsychological pathways involving stress and inflammation. Neuroscience & Biobehavioral Reviews. 2021;127:689-708.
4. Fiedler J, Eckert T, Wunsch K, Woll A. Key facets to build up eHealth and mHealth interventions to enhance physical activity, sedentary behavior and nutrition in healthy subjects - an umbrella review. BMC Public Health. 2020;20(1):1605.

5. Robert C, Erdt M, Lee J, Cao Y, Naharudin NB, Theng YL. Effectiveness of eHealth Nutritional Interventions for Middle-Aged and Older Adults: Systematic Review and Meta-analysis. Journal of medical Internet research. 2021;23(5):e15649. 6.
Ferguson T, Olds T, Curtis R, Blake H, Crozier AJ, Dankiw K, et al. Effectiveness of wearable activity trackers to increase physical activity and improve health: a systematic review of systematic reviews and meta-analyses. The Lancet Digital Health. 2022;4(8):e615-e26.
7. Kwan RYC, Salihu D, Lee PH, Tse M, Cheung DSK, Roopsawang I, et al. The effect of e-health interventions promoting physical activity in older people: a systematic review and meta-analysis. European Review of Aging and Physical Activity. 2020;17(1):7.
8. Vandelanotte C, Duncan MJ, Kolt GS, Caperchione CM, Savage TN, Van Itallie A, et al. More real-world trials are needed to establish if web-based physical activity interventions are effective. British journal of sports medicine. 2019;53(24):15534.
9. Attig C, Franke T. Abandonment of personal quantification: A review and empirical study investigating reasons for wearable activity tracking attrition. Computers in Human Behavior. 2020;102:223-37.
10. Alvarado MM, Kum HC, Gonzalez Coronado K, Foster MJ, Ortega P, Lawley MA. Barriers to Remote Health Interventions for Type 2 Diabetes: A Systematic Review and Proposed Classification Scheme. Journal of medical Internet research. 2017;19(2):e28.
11. Hutchesson MJ, Gough C, Müller AM, Short CE, Whatnall MC, Ahmed M, et al. eHealth interventions targeting nutrition, physical activity, sedentary behavior, or obesity in adults: A scoping review of systematic reviews. Obes Rev. 2021;22(10):e13295.
12. Fiedler J, Eckert T, Wunsch K, Woll A. Key facets to build up eHealth and mHealth interventions to enhance physical activity, sedentary behavior and nutrition in healthy subjects – an umbrella review. BMC Public Health. 2020;20(1):1605.
13. Skivington K, Matthews L, Simpson SA, Craig P, Baird J, Blazeby JM, et al. A new framework for developing and evaluating complex interventions: update of Medical Research Council guidance. Bmj. 2021;374:n2061.
14. Morrison LG, Muller I, Yardley L, Bradbury K. The person-based approach to planning, optimising, evaluating and implementing behavioural health interventions. The European health psychologist. 2018;20:464-9.
15. Michie S, van Stralen MM, West R. The behaviour change wheel: A new method for characterising and designing behaviour change interventions. Implementation Science. 2011;6(1):42.
16. Prochaska JO, DiClemente CC, Norcross JC. In search of how people change. Applications to addictive behaviors. The American psychologist. 1992;47(9):1102-14.
17. Michie S, Richardson M, Johnston M, Abraham C, Francis J, Hardeman W, et al. The behavior change technique taxonomy (v1) of 93 hierarchically clustered techniques: building an international consensus for the reporting of behavior change interventions. Annals of behavioral medicine : a publication of the Society of Behavioral Medicine. 2013;46(1):81-95.

18. Bergevi J, Andermo S, Woldamanuel Y, Johansson UB, Hagströmer M, Rossen J. User Perceptions of eHealth and mHealth Services Promoting Physical Activity and Healthy Diets: Systematic Review. JMIR Hum Factors. 2022;9(2):e34278. 19. Rossen J, Larsson K, Hagströmer M, Yngve A, Brismar K, Ainsworth B, et al. Effects of a three-armed randomised controlled trial using self-monitoring of daily steps with and without counselling in prediabetes and type 2 diabetes—the Sophia Step Study. International Journal of Behavioral Nutrition and Physical Activity. 2021;18(1):121.

20. Rossen J, Hagströmer M, Yngve A, Brismar K, Ainsworth B, Johansson U-B. Process evaluation of the Sophia Step Study- a primary care based three-armed randomized controlled trial using self-monitoring of steps with and without counseling in prediabetes and type 2 diabetes. BMC Public Health. 2021;21(1):1191.

21. Rossen J, Lööf H, Yngve A, Hagströmer M, Brismar K, Johansson U-B. 'This is why I'm doing a lot of exercise' — a qualitative study of participant's experiences of the Sophia Step Study. International Diabetes Nursing. 2017;14(2-3):99-104. 22. Rossen J, Yngve A, Hagströmer M, Brismar K, Ainsworth BE, Iskull C, et al. Physical activity promotion in the primary care setting in pre- and type 2 diabetes - the Sophia step study, an RCT. BMC Public Health. 2015;15:647.

23. West R, Michie S. A guide to development and evaluation of digital behaviour interventions in healthcare. London: Silverback Publishing; 2016.

24. Woldamanuel Y, Rossen J, Andermo S, Bergman P, Åberg L, Hagströmer M, et al. Perspectives on Promoting Physical Activity Using eHealth in Primary Care by Health Care Professionals and Individuals With Prediabetes and Type 2 Diabetes: Qualitative Study. JMIR Diabetes. 2023;8:e39474.

25. Lewis JR. The System Usability Scale: Past, Present, and Future. International Journal of Human–Computer Interaction. 2018;34(7):577-90.

26. Stoyanov SR, Hides L, Kavanagh DJ, Zelenko O, Tjondronegoro D, Mani M. Mobile app rating scale: a new tool for assessing the quality of health mobile apps. JMIR mHealth and uHealth. 2015;3(1):e27-e.

27. Bock BC, Marcus BH, Rossi JS, Redding CA. Motivational readiness for change: Diet, exercise, and smoking. American Journal of Health Behavior. 1998;22(4):248-58.

28. Forsberg L, Ekman S, Halldin J, Rönnberg S. The readiness to change questionnaire: reliability and validity of a Swedish version and a comparison of scoring methods. Br J Health Psychol. 2004;9(Pt 3):335-46.

29. Löve J, Moore CD, Hensing G. Validation of the Swedish translation of the General Self-Efficacy scale. Qual Life Res. 2012;21(7):1249-53.

30. Kearney JM, McElhone S. Perceived barriers in trying to eat healthier-results of a pan-EU consumer attitudinal survey. Br J Nutr. 1999;81 Suppl 2:S133-7.

31. Topp CW, Østergaard SD, Søndergaard S, Bech P. The WHO-5 Well-Being Index: A Systematic Review of the Literature. Psychotherapy and Psychosomatics. 2015;84(3):167-76.

32. Besèr A, Sorjonen K, Wahlberg K, Peterson U, Nygren A, Asberg M. Construction and evaluation of a self rating scale for stress-induced exhaustion disorder, the Karolinska Exhaustion Disorder Scale. Scandinavian journal of psychology.

2014;55(1):72-82.
33. Demerouti E, Bakker AB, Vardakou I, Kantas A. The convergent validity of two burnout instruments: A multitrait-multimethod analysis. European Journal of Psychological Assessment. 2003;19(1):12.
34. Bastien CH, Vallières A, Morin CM. Validation of the Insomnia Severity Index as an outcome measure for insomnia research. Sleep Med. 2001;2(4):297-307.
35. Socialstyrelsens frågor om levnadsvanor: Socialstyrelsen; [Available from: https://www.socialstyrelsen.se/globalassets/sharepoint-dokument/dokumentwebb/nationella-riktlinjer/levnadsvanor-fragor-om-levnadsvanor.pdf.
36. Willett W, Rockström J, Loken B, Springmann M, Lang T, Vermeulen S, et al. Food in the Anthropocene: the EAT–Lancet Commission on healthy diets from sustainable food systems. The Lancet. 2019;393(10170):447-92. 37. Yang Y, Schumann M, Le S, Cheng S. Reliability and validity of a new accelerometer-based device for detecting physical activities and energy expenditure.
PeerJ. 2018;6:e5775-e.
38. Krippendorff K. Content analysis : an introduction to its methodology. Thousand Oaks, Calif. ;: SAGE; 2013.
39. Bech P, Lindberg L, Moeller SB. The Reliable Change Index (RCI) of the WHO-5 in primary prevention of mental disorders. A measurement-based pilot study in positive psychiatry. Nordic Journal of Psychiatry. 2018;72(6):404-8.
40. Howlett N, Trivedi D, Troop NA, Chater AM. Are physical activity interventions for healthy inactive adults effective in promoting behavior change and maintenance, and which behavior change techniques are effective? A systematic review and meta-analysis. Transl Behav Med. 2019;9(1):147-57.
41. Ellervik C, Kvetny J, Christensen KS, Vestergaard M, Bech P. Prevalence of depression, quality of life and antidepressant treatment in the Danish General Suburban Population Study. Nordic Journal of Psychiatry. 2014;68(7):507-12.
42. Morton K, Dennison L, May C, Murray E, Little P, McManus RJ, et al. Using digital interventions for self-management of chronic physical health conditions: A metaethnography review of published studies. Patient education and counseling. 2017;100(4):616-35.
